# The role of nocturnal fishes on coral reefs: A quantitative functional evaluation

**DOI:** 10.1002/ece3.9249

**Published:** 2022-08-26

**Authors:** William P. Collins, David R. Bellwood, Renato A. Morais

**Affiliations:** ^1^ Research Hub for Coral Reef Ecosystem Functions, College of Science and Engineering and ARC Centre of Excellence for Coral Reef Studies James Cook University Townsville Queensland Australia

**Keywords:** biomass, diel habits, diurnal fishes, ecosystem function, nocturnal fishes, productivity

## Abstract

The ecological functions of nocturnal coral reef fishes are poorly known. Yet, nocturnal resources for coral reef consumers are theoretically as abundant and productive, if not more so, than their diurnal counterparts. In this study, we quantify and contrast the energetic dynamics of nocturnal and diurnal fishes in a model coral reef ecosystem, evaluating whether they attain similar levels of biomass production. We integrated a detailed dataset of coral reef fish counts, comprising diurnal and nocturnal species, in sites sheltered and exposed to wave action. We combined somatic growth and mortality models to estimate rates of consumer biomass production, a key ecosystem function. We found that diurnal fish assemblages have a higher biomass than nocturnal fishes: 104% more in sheltered sites and 271% more in exposed sites. Differences in productivity were even more pronounced, with diurnal fishes contributing 163% more productivity in sheltered locations, and 558% more in exposed locations. Apogonidae dominated biomass production within the nocturnal fish assemblage, comprising 54% of total nocturnal fish productivity, which is proportionally more than any diurnal fish family. The substantially lower contributions of nocturnal fishes to biomass and biomass production likely indicate constraints on resource accessibility. Taxa that overcome these constraints may thrive, as evidenced by apogonids. This study highlights the importance of nocturnal fishes in underpinning the flow of energy and nutrients from nocturnal resources to reef communities; a process driven mainly by small, cryptic fishes.

## 1 INTRODUCTION

Daytime (diurnal) ecological studies far outpace night‐time (nocturnal) studies (Gaston, 2019; Park, 1940). This diurnal research bias in ecology, likely driven by a plethora of logistical challenges, is exacerbated in submerged aquatic systems where light is naturally scarce, and where organisms must be studied in situ. Coral reefs typify this disparity resulting in a fragmentary understanding of the ecological functions performed by coral reef organisms during the nocturnal period. This is true even for major biological components of these high‐diversity systems, such as reef fishes (see Fox & Bellwood, 2011).

Understanding community structure is one of the first steps toward a deeper understanding of ecosystem functioning. Community structure offers insights into the relative importance of differing groups of organisms within the ecosystem, and is often used to quantify changes in ecosystems through time (e.g., Bellwood et al., 2006; Jackson & Blois, 2015; Stevens, 2009; Syms & Jones, 2000). Reef fishes are a conspicuous, species rich, morphologically diverse, and abundant group of organisms on coral reefs (Bellwood et al., 2019; Brandl et al., 2019). Because of the diverse roles they play in coral reef ecosystems, reef fishes have been a major focus of coral reef ecology (Mora, 2015; e.g., Morais, Connolly, & Bellwood, 2020; Plass‐Johnson et al., 2015; Streit et al., 2019; Tebbett et al., 2017). However, despite the numerous studies characterizing reef fish community structure and/or composition (e.g., Bellwood et al., 2006; Hémery & McClanahan, 2005; Kane & Tissot, 2017; Syms & Jones, 2000), explicit evaluations of temporal partitioning of reef communities into diurnal and nocturnal communities are surprisingly rare. For example, on the Great Barrier Reef, there are no quantitative comparisons of diel whole reef fish community patterns, with only a few studies focusing on commercially important fisheries target species (Cappo et al., 2004; Newman & Williams, 1995, 2001). Therefore, we still lack a comprehensive quantitative comparison of the relative importance of nocturnal and diurnal reef fishes in the overall community structure and, most importantly, in their relative contribution to major ecosystem functions, especially biomass production.

Based on the presence of potential food resources one may hypothesize that the biomass and productivity of nocturnal feeding fishes would be comparable to their diurnal counterparts. Sessile primary producers and invertebrates do not change in abundance between day and night, and even in photosynthetically active taxa, key nutritional components (protein) are likely to remain consistent (Zemke‐White et al., 2002). Similarly, motile invertebrates and small fishes also remain on the reefs at night. But it is in the plankton that changes are most likely, with emergent and migratory nocturnal plankton boosting nocturnal planktonic communities (Hobson & Chess, 1979; Kramer et al., 2013; Yahel et al., 2002, 2005). Nocturnal planktonic communities are more diverse and abundant than corresponding diurnal planktonic communities (Carleton & Hamner, 2007; Hammer, 1981; Hobson & Chess, 1979; Sorokin & Sorokin, 2009; Yahel et al., 2005). One may therefore expect to find more fish‐based production, especially in planktivores, at night.

**FIGURE 1:**
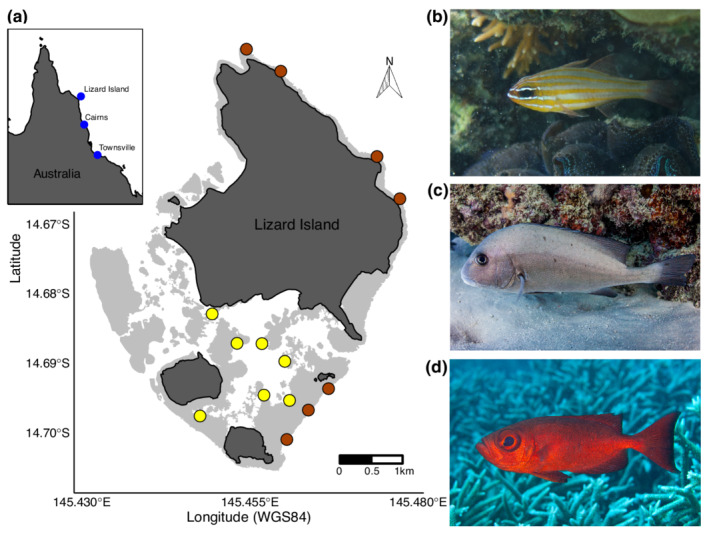
(a) Map of Lizard Island, in the northern Great Barrier Reef. Sample sites are highlighted with yellow dots representing the “sheltered” sites and brown dots representing “exposed” sites. Photos depict examples of nocturnal coral reef fishes. (b) the cardinalfish, *Ostorhinchus cyanosoma* (Apogonidae). (c) Sweetlips, *Diagramma pictum* (Haemulidae). (d) Bigeye, *Priacanthus hamrur* (Priacanthidae). c and d photos by Victor Huertas.

The nocturnal period is a known time of activity for many reef predators (Connell, 1998; Danilowicz & Sale, 1999), particularly, for mobile fish species that travel off the reef to feed at night (Burke, 1995; Nagelkerken & van der Velde, 2004). However, feeding under low light conditions is challenging, particularly for predators of elusive or camouflaged prey (e.g., other fishes or planktonic crustaceans). Nocturnal feeding is thus normally limited to species with morphological and/or behavioral traits that allow them to feed in low light conditions (Goatley & Bellwood, 2009; Schmitz & Wainwright, 2011). Because of these physiological challenges, most of the visually oriented plankton feeders that dominate reefs during the day are absent during the night (Hobson, 1965; Hobson & Chess, 1979). This raises the key issue of presence versus availability: To what extent does prey presence equate to prey availability? One way of disentangling the relative availability of nocturnal versus diurnal prey to fish consumers is by measuring the biomass production of nocturnal and diurnal fish communities. If prey presence is the primary factor they should be similar; if nocturnal constraints restrict availability they are likely to differ. These relationships may also be context‐dependent, as there is evidence of spatial variation in fish productivity (Morais & Bellwood, 2019). By assessing locations that are both sheltered and exposed to wind and wave action (Figure 1), we can address the potential of spatial variation in hydrodynamics to shape differences in biomass and productivity.

Fish communities are typically evaluated or inferred based on numeric variables such as the abundance of individuals or species richness. However, while these variables tell us which individuals and species are present or absent from a location, that is, how prevalent they are in the community, they convey little information with regard to their role in the ecosystem. To explore the functional contribution more directly, we assess the nocturnal versus diurnal community structure through more functionally relevant metrics such as biomass (i.e., the cumulative weight of organisms) and productivity (i.e., the cumulative net biomass produced) (cf. Morais & Bellwood, 2020). We thus provide a detailed comparison of the biomass and productivity of diurnal versus nocturnal reef fishes within a coral reef system, while simultaneously exploring differences between exposed and sheltered locations—an important determinant for community structure which may also shape the relative intensity of ecological functions (Bronstein & Loya, 2014; Davis et al., 2021; Depczynski & Bellwood, 2005; Fulton & Bellwood, 2005; Valenzuela et al., 2021). By scaling individual contributions to energetic or nutrient flows, we provide insights into the role of diurnal versus nocturnal organisms in storing or moving energy or material within or between ecosystems: key reef‐scale ecosystem processes (Bellwood et al., 2019).

## 2 MATERIALS AND METHODS

### 2.1 Data collection

To compare the relative contributions of diurnal and nocturnal fishes to community‐level production of biomass, we carried out 75 underwater visual surveys using SCUBA on the mid‐shelf reefs at Lizard Island, in the northern Great Barrier Reef (GBR) (Figure 1a) between April 2017 and December 2018. All surveys were conducted between 10:00 and 15:00 h by the same experienced observer (R. Morais), to keep observer bias consistent across counts. The surveys were specifically designed to encompass all visually apparent fishes, including nocturnal species (following Ackerman & Bellwood, 2000; Morais & Bellwood, 2019). Each survey consisted of four overlapping transects, each transect focusing on a different set of species from the total fish assemblage, to help maximize the fish species detected within the survey area.

Our UVC method was designed to include components of the fish community that are often missed from traditional UVCs (Ackerman & Bellwood, 2000). Our method (based on the findings of Ackerman & Bellwood) was specifically designed to ensure we recorded as many nocturnal species as possible, especially apogonids. However, it is inevitable that a number of cryptobenthic fishes, predominantly gobies and other small cryptobenthic forms, will have been missed. However, these smaller cryptobenthic fishes contribute very little to biomass and only a small amount to biomass production on reefs (Brandl et al., 2019; Morais & Bellwood, 2019). Furthermore, most if not all are likely to be diurnal. The nocturnal proportion of the cryptobenthic community (driven greatly by Apogonidae) is well retained in combined UVC strip censuses when compared with results from rotenone stations (the most effective way to sample cryptobenthic communities; Ackerman & Bellwood, 2000, 2002). Biomass and its production is often dominated by planktivores, inconspicuous medium‐sized species or relatively small, but conspicuous, species (Brandl et al., 2019; Morais et al., 2021; Morais & Bellwood, 2019). Unlike almost all other UVCs, our methods include counts specifically aimed at these fishes. We do not consider immigration, emigration, or recruitment; we record the individuals visually apparent at the time of the survey.

Overall, our surveys provide a snapshot of the visually apparent reef fishes on a coral reef. The nocturnal fish counts are, if anything, likely to be slightly overestimated (as noted below), while the diurnal counts are likely to be slightly conservative (due to missing small cryptobenthic taxa). For further information on the methods, see Appendix S1.

### 2.2 Defining nocturnal reef fishes

To classify the diel habits of all the fish species in the surveys, information on each species was collated using the One Search engine on the James Cook University library system, with the search string “*Genus species*” AND “nocturnal” OR “diurnal” OR “diel.” Relevant research papers were checked for evidence of quantification of diel habits. Species level diel habits were then recorded as: diurnal, nocturnal, or both. To account for the bias in research effort (diurnal vs. nocturnal studies), we only considered a species in this search as exclusively diurnal, nocturnal, or both, if there was clear evidence that the species had been monitored during both the day and night. For this study, species which were considered as crepuscular feeders were included in the “both” category. If there was no evidence (or in most cases no studies) looking at their activity during both time periods, we referred to Fishbase (Froese & Pauly, 2021), Randall et al. (1998) and/or (Myers, 1999). When no explicit mention of diel habits was made for a species in any of these references, the typical values for the genus or family level behavior were used in conjunction with expert assessments (from the Research Hub for Coral Reef Ecosystem Functions at JCU). Expert assessments were based on combined prior experience amounting to several 1000 h of diving. The final diel classification of all species referred to in this study is given in Table S1. As this study focuses on the habits of fishes active during the night, we consider “nocturnal” fishes herein as those that are either exclusively active during the night or facultative nocturnal fishes (inc. crepuscular) which may be active during the day and night (the fishes considered as “both”). This meant that species which were considered as “both” were pooled with the nocturnal fishes for the analysis. Overall, the “nocturnal” fishes included herein encompasses obligate nocturnal fishes and a range of others that may feed at night (inc. facultative nocturnal fishes and crepuscular feeders).

### 2.3 Estimating standing biomass and productivity from underwater fish surveys

In order to estimate the standing biomass and productivity of fishes from the visual surveys, we followed the methods of Morais and Bellwood (2020), with further details outlined in Appendix S1. In short, the weight of individuals was calculated based on species‐specific length‐weight conversion factors as compiled by FishBase (Froese, R. and Pauly, D. Froese & Pauly, 2021). Individual weights were then summed across all individuals of diurnal and nocturnal species to output their standing biomass. Productivity was derived from the balance between the estimated cumulative somatic growth and mortality of all individuals. Growth was assumed to follow a Von Bertalanffy Growth model and was estimated from the derived Kmax coefficient and species maximum size (Morais and Bellwood 2018). Mortality was simulated stochastically based on the probability of mortality of individual fishes (Morais & Bellwood, 2020), which was based on the estimated instantaneous mortality parameter “M”. Simulations used a Bernoulli distribution to assign the fate (survival or mortality) for each individual after 1 day, with the total productivity being the cumulative somatic growth of surviving individuals. Stochastic mortality simulations were repeated over 1000 iterations. Biomass and productivity were calculated for each sample (survey) by summing individual body masses and average productivities of all fishes in that survey. Finally, biomass and productivity were averaged across samples for sheltered and exposed locations, and for nocturnal and diurnal representatives of each family. Biomass is presented as tonnes per hectare (t ha^−1^) and productivity is presented as grams per 100 m^2^ per day (g 100 m^−2^ day^−1^). A more detailed description of all these procedures can be found in Appendix S1 and Morais and Bellwood (2020).

### 2.4 Data analyses

All statistical analyses were carried out using R (R Core Team, 2021). To test for differences in community biomass and productivity between site exposure types and diel habits (nocturnal vs. diurnal), as well as the biomass and productivity of Apogonidae between site exposure types, we used Bayesian generalized linear mixed effect models. Each model included either biomass or productivity as the response variable. For each model, both habits (diurnal vs. nocturnal) and site type (exposed vs. sheltered) were used as explanatory variables, and survey number (sample) nested within site was included as a random effect. These models were developed with the No‐U‐Turn Markov Chain Monte Carlo (MCMC) sampler in Stan via “rstan” (Stan Development Team, 2020) and “rstanarm” (Goodrich et al., 2020) in R. For each model, we used 5000 iterations per chain in a total of four chains, including a 50% burn‐in. The distribution, priors, and diagnostics used in these models can be found in Appendix S1.

Non‐metric multidimensional scaling (NMDS) of Bray–Curtis dissimilarity on square‐root and Wisconsin double‐standardized data were used to inspect the taxonomic (family) composition of the productivity and biomass of nocturnal fishes and how they varied between exposed and sheltered sites. These were performed using the “metaMDS” function from the “vegan” (Oksanen et al., 2020) package in R. Details are outlined in Appendix S1.

## 3 RESULTS

### 3.1 Biomass

**FIGURE 2:**
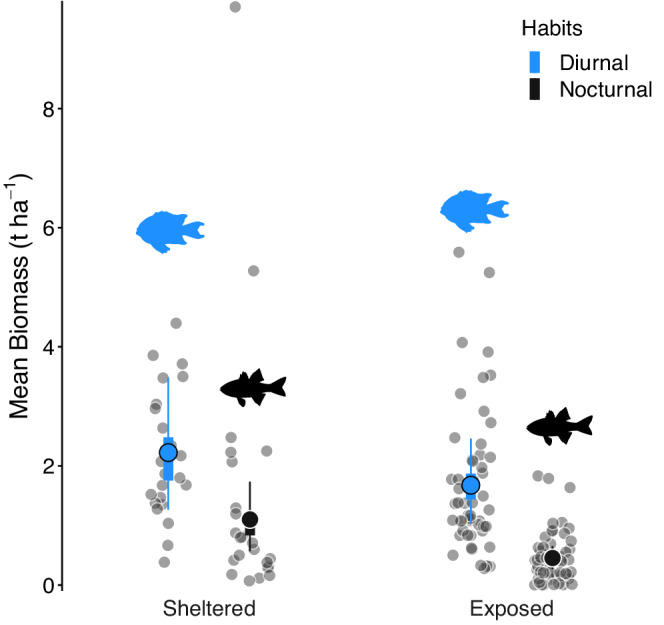
Diurnal (blue) and nocturnal (black) fish biomass estimates. Gray dots represent the raw datapoints. Large, colored dots represent the median biomass from the posterior distribution. Thick bars represent the 50% credible intervals, and the thin bars represent the 95% credible intervals.

The standing stock of diurnal reef fishes was higher than nocturnal reef fishes in both sheltered and exposed locations (Table 1). Across MCMC samples, the diurnal fish biomass was greater than nocturnal fish biomass 91% of the time. In sheltered sites, diurnal fish biomass (median: 2.22 t ha^−1^, HPD: 1.26–3.49; Figure 2; Table 1) exceeded nocturnal fish biomass (median: 1.10 t ha^−1^, HPD: 0.57–1.74; Figure 2; Table 1), comprising on average 104% more biomass (β_DS/NS_ = 2.04, HPD: 1.03–3.31). In exposed sites, these differences were even clearer, as diurnal fishes comprised 271% more biomass than nocturnal fishes (β_DE/NE_ = 3.71, HPD: 2.44–5.05; Figure 2).

**TABLE 1:** Posterior estimates (mean and medians) with 95% high posterior density intervals (HDI) from Bayesian generalized linear models comparing the biomass and productivity of nocturnal reef fishes.

Metric	Habits	Site type	Mean	Median (lower ‐ upper 95% HDI)
Biomass (t ha^−1^)	Diurnal	Sheltered	2.30	2.22 (1.26–3.49)
		Exposed	1.71	1.68 (1.04–2.46)
	Nocturnal	Sheltered	1.14	1.10 (0.57–1.74)
		Exposed	0.46	0.45 (0.27–0.65)
Productivity (g 100 m^−2^ day^−1^)	Diurnal	Sheltered	4.24	4.11 (2.48–6.31)
		Exposed	3.04	2.99 (1.90–4.22)
	Nocturnal	Sheltered	1.62	1.58 (0.88–2.42)
		Exposed	0.46	0.45 (0.30–0.64)

Site exposure, however, had a weaker effect than diel habits (Figure 2). The biomass of diurnal fishes, for example, was only 33% higher on sheltered locations compared to exposed locations, yet this involved large variability (β_DS/DE_ = 1.33, HPD: 0.59–2.40), with the probability of this contrast (Biom_DS_ > Biom_DE_) being only 82%. Site type had a stronger effect for the biomass of nocturnal fishes, with the median nocturnal fish biomass at sheltered sites being 142% (β_NS/NE_ = 2.42, HPD: 1.12–4.55) greater than exposed sites, with a probability of 99.1%.

### 3.2 Productivity

**FIGURE 3:**
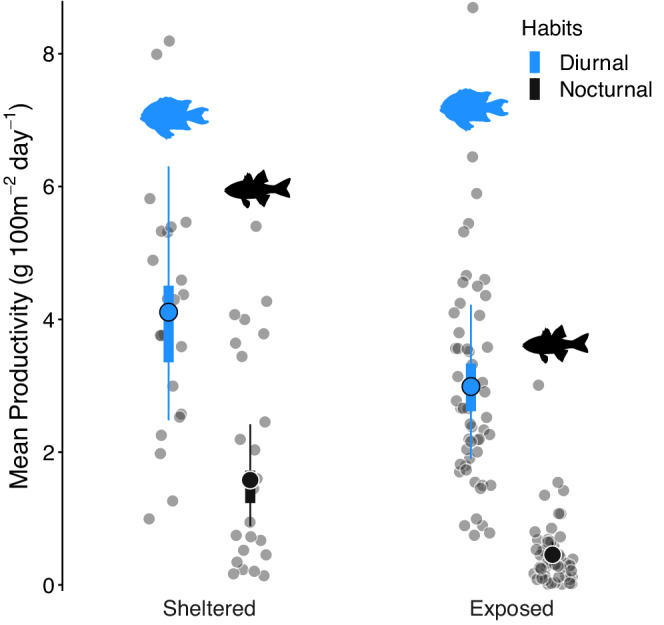
Diurnal (blue) and nocturnal (black) fish productivity estimates. Gray dots represent the raw datapoints. Large, colored dots represent the median productivity from the posterior distribution. Thick bars represent the 50% credible intervals, and the thin bars represent the 95% credible intervals.

The same general trends were found for productivity, except that the magnitude of the differences was much greater (Figure 3). Overall, the diurnal fish productivity was higher than nocturnal fish productivity in both sheltered and exposed locations (Table 1).

In sheltered sites, the median diurnal fish productivity (4.11 g 100 m^−2^ day^−1^, HPD: 2.48–6.31; Figure 3; Table 1) was 163% (β_DS/NS_ = 2.63, HPD: 1.53–4.08) greater than the median nocturnal fish productivity (1.58 g 100 m^−2^ day^−1^, HPD: 0.88–2.42; Figure 3; Table 1). In exposed sites, the differences between median diurnal productivity (2.99 g 100 m^−2^ day^−1^, HPD: 1.90–4.22; Figure 3; Table 1) and median nocturnal fish productivity (0.45 g 100 m^−2^ day^−1^, HPD: 0.30–0.64; Figure 3; Table 1) were even more contrasting, with diurnal fishes supporting 558% (β_DE/NE_ = 6.58, HPD: 4.70–8.79) more productivity than nocturnal fishes.

As with biomass, site exposure also had a weaker effect than diel habits on productivity (Figure 3; Table 1). Diurnal fish productivity in sheltered sites was 38% higher than in exposed locations, yet with large variability (β_DS/DE_ = 1.38, HPD: 0.67–2.38). The probability of this contrast (Prod_DS_ > Prod_DE_) was 87%. However, as in biomass, there was a strong effect of site exposure on median nocturnal fish productivity, with sheltered sites having, on average, 250% (β_NS/NE_ = 3.50, HPD: 1.66–5.92) more nocturnal fish productivity than exposed sites, with ~100% probability.

### 3.3 Community composition: Nocturnal productivity

**FIGURE 4:**
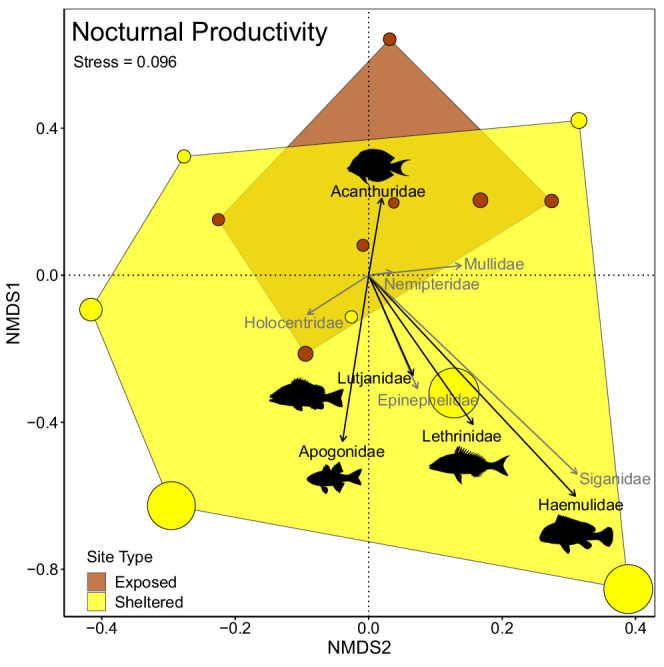
Site level NMDS plots of (exposed in brown, sheltered in yellow) nocturnal species which were grouped into a family level. The figure shows only the top 10 nocturnal fish families (based on their contributions to productivity). The top five families are in represented by the color black and the top 6–10 families are in gray. Dot size represents the relative productivity of each site (diameter scaled to productivity).

The multivariate family structure of nocturnal biomass and productivity, exemplified by the NMDS plots, showed a higher variability at sheltered sites compared to exposed sites (Figure 4; Figure S1). Overall, sites with high productivity tended to be sheltered, with site differences being driven mainly by the productivity of Siganidae, Haemulidae, Lethrinidae, Apogonidae, Lutjanidae, and Epinephelidae (Figure 4).

**FIGURE 5:**
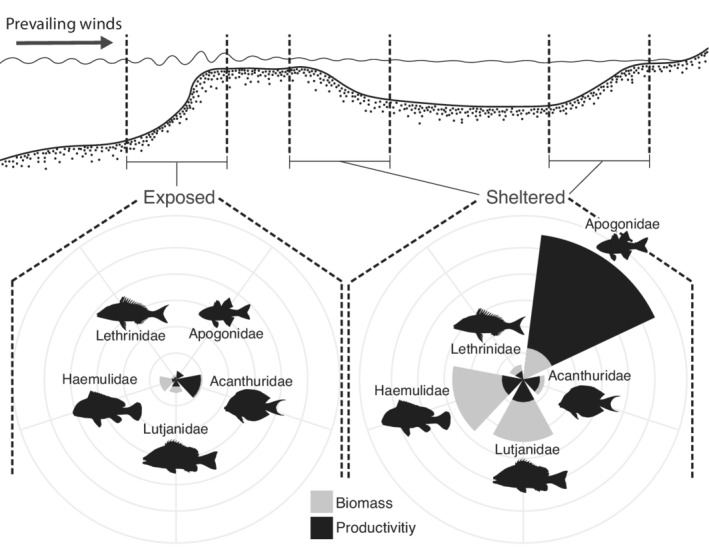
Reef schematic showing a cross‐sectional depiction of a reef. The exposed and sheltered locations are highlighted. The relative biomass (gray) and productivity (black) of the top five nocturnal fish families across exposed and sheltered locations (only the nocturnal species from each family are included). For values see Table S2.

Looking in more detail at the productivity of individual nocturnal fish families, two clear patterns were detected. First, family‐specific biomass and productivity (of the top five nocturnal families based on productivity) were smaller in exposed compared to shelter habitats, in all families except Acanthuridae (Figure 5; Figure S2; Table S2). Second, there was an overwhelming contribution of Apogonidae productivity in sheltered locations (Figure 5; Figure S2). Furthermore, because total productivity was much higher in sheltered sites, the overall contribution of apogonids to total productivity, regardless of exposure type, was larger than all other families, comprising 54% of all nocturnal fish productivity in the study (Figure S2, Table S2).

**FIGURE 6:**
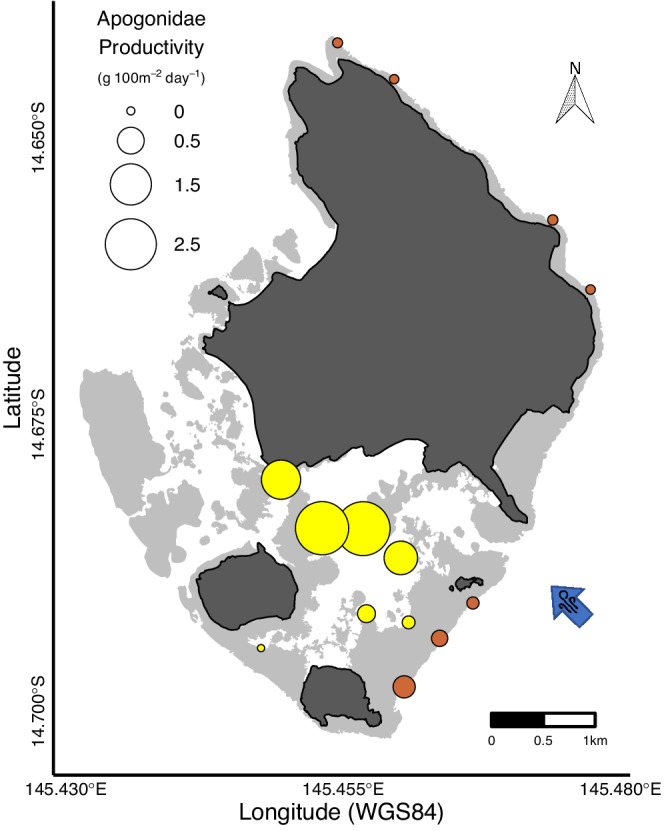
Mean estimates of apogonid productivity at the sample sites (exposed in brown and sheltered in yellow) around Lizard Island. Light gray shades represent the reef contour and dark gray shades represent land. The blue arrow indicates the prevailing south easterly wind direction.

The productivity of Apogonidae at sheltered sites was 1300% greater (β_S/E_ = 14, HPD: 0.47–59.6) than their productivity at exposed sites (Figures 5 and 6; Figure S2). Although there was very large variability, the probability of this contrast was 99.5%. This variability was manifested spatially, with sites in the inner part of the lagoon having the highest productivities, and sites on the outer part of the lagoon having productivity similar to exposed sites (Figure 6).

## 4 DISCUSSION

By combining surveys of whole fish communities and a recently developed method for estimating biomass production, we were able to estimate the contributions of both diurnal and nocturnal fishes to community level consumer productivity on a coral reef in the largest reef system in world, the Great Barrier Reef. We found that diurnal fishes produced much more biomass than nocturnal fishes, with this difference being particularly pronounced in exposed sites. This finding contrasts with expectations that the greater (theoretical) availability of resources during the nocturnal period has the potential to allow nocturnal consumers to attain higher biomass and productivity. This suggests that the presence of nocturnal resources is not the main limiting energetic factor. These results, therefore, provide a window into the trophodynamics of reefs at night. In doing so, they emphasize a likely balance between the opportunities provided and constraints imposed when exploiting abundant, yet potentially inaccessible, resources. Below, we explore, in detail, these main findings and their implications.

### 4.1 Biomass storage versus production in diurnal versus nocturnal fishes

It is well established in ecology that organisms can only persist in locations where there are sufficient resources for survival (Begon et al., 2005; Odum & Barrett, 2005). At first glance, it would be tempting to assume that the results we have presented could be driven by fewer resources at night compared to the day. However, there is evidence to suggest that there is as much, if not more, resources available during the night compared to the day. For example, sessile primary producers (i.e., macroalgae, algal turfs, corals) do not go anywhere at night and other common fish prey, such as benthic invertebrates remain on reefs during the night. Furthermore, it has been shown that the planktonic and motile benthic invertebrate communities on coral reefs are more diverse and/or abundant at night compared to the day (e.g., Hobson & Chess, 1979; Kramer et al., 2013; Yahel et al., 2002, 2005; Zapata‐Hernández et al., 2021). Core to this high resource availability at night are the subsidies introduced: the emergence of resident benthic plankton and the vertical migration of deeper water pelagic plankton to shallower habitats that include reefs (Hammer, 1981; Hobson & Chess, 1979). In summary, there is evidence suggesting that the resource pool available for coral reef fishes is the same or greater at night compared to the day. However, what appears to change is their relative availability for consumption by the fishes. The most obvious conclusion is that there may be difficulties associated with accessing some of these resources (i.e., the presence of functional constraints). These functional limitations may be reflected in the morphological features of diurnal and nocturnal reef fish assemblages.

Nocturnal coral reef fishes tend to possess a suite of “standard” morphological traits: a combination of greater relative eye diameter and greater relative horizontal mouth gape compared to most other reef fish ecomorphotypes (Goatley & Bellwood, 2009; Mihalitsis & Bellwood, 2019; Schmitz & Wainwright, 2011). These morphological differences may allow nocturnal fishes to locate and feed on prey in low light conditions (Luehrmann et al., 2020; Shand, 1997; Warrant, 2004). From the fossil record, it seems that this combination of morphological traits became prevalent in the Eocene, 50 million years ago (Goatley et al., 2010). Moreover, throughout the evolution of marine fishes, only a limited number of strictly nocturnal reef fish lineages have arisen (Rabosky et al., 2018; Siqueira et al., 2020). The fact that “strict” nocturnality has appeared relatively few times in the marine fish tree of life adds support to the suggestion that the predominance of diurnal versus nocturnal fish biomass and productivity is related to the challenges of accessing reef resources at night. Teasing apart the potential causes of variation in nocturnal biomass and productivity between exposed and sheltered locations may help to explain the basis of these constraints.

### 4.2 Habitat as a driver of fish productivity & biomass

Although both diurnal and nocturnal reef fishes had less biomass and lower productivity on exposed sites compared to sheltered ones, these differences were particularly pronounced for nocturnal fishes. Given that the exposed and sheltered sites were part of the same reef system, this raises the question: what are the major factors distinguishing these habitat types, and how could they be influencing the biomass and productivity of nocturnal fishes? There are three key aspects that need to be considered: water movement, depth, and prey availability.

In the study location, exposed sites are subject to the dominant south eastly winds. As such, they experience much greater wave action and, presumably, also current motion than the sheltered sites (Fulton & Bellwood, 2005; Jokiel & Morrissey, 1993). Hydrodynamics could be, thus, influencing the distribution of nocturnal fishes in multiple ways. First, it could directly affect habitat occupancy and, with it, feeding ability. Occupying and feeding in high‐energy environments requires morphological traits enabling fish to cope with these conditions, often dependent on fin morphology (Fulton et al., 2005). To date, the major morphological distinctions among diurnal versus nocturnal fishes have been found in predatory fishes (e.g., Goatley & Bellwood, 2009; Mihalitsis & Bellwood, 2019; Schmitz & Motani, 2010; Schmitz & Wainwright, 2011). Nocturnal fish morphology is typified by *Myripristis sp*., the Soldierfishes, which have large eyes and large relative mouth gapes. Nocturnal piscivores also have intermediate fin aspect ratios between those of pelagic and diurnal benthic piscivores (Mihalitsis & Bellwood, 2019). As fin aspect ratio has been closely linked to sustained swimming speeds (Fulton et al., 2005), this suggests that nocturnal piscivores may have only limited swimming competency in high‐energy environments (Mihalitsis & Bellwood, 2019), potentially limiting their capacity to occupy these habitats, or conferring higher energetic costs. A similar constraint may also apply to other nocturnal fishes (such as apogonids) which also tend to have moderate to low aspect ratio fins (holocentrid's caudal fins are deeply forked which may reflect a more intermediate swimming capacity).

Compared to sheltered locations, exposed locations are also situated in, or closer to, deeper water (Figure 1). Water filters light, while suspended sediments scatter light (Mayerhöfer et al., 2020). Increasing water column depth, therefore, reduces light availability. An important implication of this is that, at night (where the initial input of light is low), the light attenuates quicker with increased depth, and therefore light energy changes significantly with small changes in depth (Abdelrhman, 2017). In essence, it is possible that shallower and presumably better‐illuminated feeding habitats in sheltered locations would enhance the capacity of nocturnal fishes to detect and/or capture prey compared to deeper and presumably less well‐illuminated feeding habitats in exposed locations.

Evidence with which to test this hypothesis is, however, scarce. Using flume chamber feeding experiments that simulated different levels of natural illuminance, Holzman and Genin (2003) have shown that *Apogon annularis*, still had significant feeding success at light levels equivalent to 18 m depth on a moonless night and 47 m on full moon nights (Holzman & Genin, 2003). Job (1999) likewise showed that pre‐settlement apogonid larvae would be able to feed down to 15 m in full moonlight intensities. However, even the most sensitive larvae would be incapable of visually mediated feeding at light intensities around the new moon (Job, 1999). In the present study, the depth of the reef‐sand interface (i.e., reef base) in the exposed sites exceeds 18 m (Leon et al., 2013), while water turbidity is likely to be much higher than the simulated clear water reef conditions in the Red Sea from Holzman and Genin (2003). Therefore, it is possible that the depth of sandy substrata may be a factor limiting the occupancy and thus the productivity of nocturnal fishes, via decreased feeding success, although this scenario remains highly speculative.

Finally, it is also possible that the observed differences in nocturnal fish productivity between exposed and sheltered locations reflect not limitations on predatory features of nocturnal fishes, but on the availability of their prey. Most nocturnal fishes are planktivorous, feeding on the larger reef‐resident emergent plankton (Carleton & Hamner, 2007; Hobson, 1965, 1991; Holzman & Genin, 2003; Marnane & Bellwood, 2002). It, therefore, seems likely that the distribution of nocturnal fishes would match that of their prey. The larger emergent plankton has been shown to be more associated with the soft sediment environment within lagoons and sand flats on coral reefs including around Lizard Island (Alldredge & King, 1977). These sandy areas near reefs are known to concentrate reef detritus, which presumably composes an important part of the diet of these emergent plankton (Carleton & McKinnon, 2007). It is also possible that these resident plankton could be actively avoiding high current areas on the edge of reefs which may pose increased risk of displacement (cf. Hobson, 1991).

Whether influenced directly (i.e., waves, current, or lack of light limiting fish feeding) or indirectly (i.e., reduced prey due to waves, current, or light), exposed sites had lower productivity and biomass of diurnal and, particularly, of nocturnal fishes. It may be that reduced benthic productivity on the sand of exposed reefs (Alldredge & King, 1977; Carleton & McKinnon, 2007), reduced visual detectability (Holzman & Genin, 2003; Job, 1999) and increased cost of foraging (Fulton et al., 2005; Mihalitsis & Bellwood, 2019) act in synergy to limit nocturnal fish productivity in exposed locations. It should also be noted that Lizard Island has been exposed to severe disturbances that have removed much of the coral cover in exposed locations. The observed patterns must, therefore, be placed in the context of low‐coral cover in exposed locations, and the broader generality of our observations and interpretation requires further investigation.

### 4.3 Apogonidae: Their role in nocturnal productivity

Our community‐level evaluation of biomass and productivity reflects a well‐known division in foraging activity, diurnal versus nocturnal. However, our data also revealed a division within nocturnal fishes. Most nocturnal families detected are relatively large‐bodied with a moderate contribution to both total community biomass and productivity (e.g., Acanthuridae, Lutjanidae, Haemulidae, and Lethrinidae). In marked contrast, Apogonidae are small‐bodied, have low total community biomass, yet delivered the highest productivity. This family alone comprised 54% of all nocturnal fish productivity, more than all other families combined. Apogonidae also showed a very clear distinction between exposed and sheltered locations, in contrast to the other families, with apogonids in sheltered locations having 1300% more productivity than in exposed locations (Figures 5 and 6; Figure S2). Apogonids, thus, appear to be an extremely important part of the nocturnal fish assemblage, with a dominant role in driving the variation in nocturnal fish productivity between sheltered and exposed sites. The exact reason for this distinction between sheltered and exposed sites, however, remains unclear. Results from various homing studies have shown that site fidelity in apogonids can be driven by both social preferences and habitat, depending on the species (Gardiner & Jones, 2005, 2010; Rueger et al., 2016). When assessing microhabitat types for apogonid resting sites, Gardiner and Jones (2005) showed that nine of their 10 study species were strongly associated with live scleractinian coral cover. In contrast to these results, Wismer et al. (2019) documented at Lizard Island a 43.1% reduction in total live coral cover between 2016 and 2018 that was paralleled by an 8600% increase in apogonid recruit and juvenile abundances, and a 178.6% increase in adult apogonid abundance. However, without species‐level identification, we cannot say whether these results contradict prior reports of reliance on live coral cover, or a possible switch between species with a high versus low reliance on live corals.

Interestingly we found a divide in apogonid production within the sheltered sites (Figure 6). The four innermost sheltered sites had very high apogonid productivity whereas the other three had productivity levels similar to the exposed sites (Figure 6). For this reason, it seems likely that the major factors, as outlined previously (wave action, prey availability, and light), that distinguish exposed versus sheltered locations, are acting on a different scale for apogonids. Due to their small body size and reduced swimming ability, life in high‐energy locations would be particularly energetically demanding for apogonids with poor swimming abilities (cf. Stobutzki & Bellwood, 1997). It would also increase predation risks for apogonids, making the exposed locations far from ideal resting sites. However, understanding the divide within sheltered locations would require a much more detailed understanding of the reefs on a finer scale.

Our findings suggest the existence of two major functional groups of nocturnal fishes. The first group incorporates those generally larger in body size with a high standing biomass and relatively low productivity. The second group includes those smaller in body size which have a low standing biomass and high productivity, typified by the apogonids. A clear path forward in detailing these functional roles will require an understanding of their spatial context. Apogonids typically feed over the shallow sandy lagoon substrata close to the reefs (Marnane & Bellwood, 2002). Given their outstanding reported homing ability (Gardiner & Jones, 2016; Marnane, 2000), it is not impossible that these fishes could be traveling hundreds of metres to feed each night, as has been reported for pempherids, another family of small nocturnal reef fishes (Koeda et al., 2021). If this is the case, the high productivity of apogonids could represent a significant source of spatial subsidies underpinning energy and nutrient flows from off‐reef locations to coral reefs (Morais et al., 2021).

Compared to apogonids, even less is known about the functional movement of larger nocturnal fishes on coral reefs. We know from acoustic tracking studies that these larger, high biomass families have the capacity to move great distances (Hitt, Pittman, & Brown, 2011; Hitt, Pittman, & Nemeth, 2011), however, the locations where they feed or occupy at night, in general, are poorly known, and may not be obvious from our knowledge of their diurnal habits. A clear example is the rabbitfish *Siganus lineatus*, which has been found to include sharp variations in diel activity between groups of individuals only a few kilometers apart (Fox & Bellwood, 2011). On top of that, some of these larger nocturnal fish families, such as Lutjanidae and Lethrinidae, are fisheries‐target groups on coral reefs in the Indo‐Pacific (Hutchings et al., 2019; Newman & Williams, 2001). A better knowledge of their functional role and spatial usage would help inform fisheries management and potential conservation efforts.

In summary, this study provides the first quantitative, functional, comparison of nocturnal and diurnal fishes on a typical coral reef in the largest reef system on Earth. These findings further our understanding of the energetic landscape of day versus night in coral reef systems. Despite similar availability of resources between diurnal and nocturnal periods, we have shown that diurnal fishes have much greater contributions to community‐level biomass production compared to nocturnal fishes. These results highlight the potential evolutionary and ecological constraints of a nocturnal lifestyle and unveiled a strong spatial variation in the productivity and biomass of nocturnal fishes, which is highest in shallow sheltered locations. Within the nocturnal fishes, we revealed two distinct energetic strategies. Firstly, the smaller nocturnal fishes with low biomass and high productivity, driven mainly by apogonids, displaying energetic strategies which facilitate a high level of energy movement through the system. And secondly the larger nocturnal fishes with high biomass and low productivity, which store/retain energy and contribute very little to the production of new biomass. These findings set the scene and provide a steppingstone for studying energetic pathways in nocturnal fishes. They raise many questions regarding the spatial footprint of these energy transactions: where do highly productive nocturnal fishes feed, what are the constraints of feeding in nocturnal systems, and what are these fishes are feeding on? All of which would provide us with a more holistic understanding of coral reef ecology.

## AUTHOR CONTRIBUTIONS

**William Peter Collins:** Conceptualization (equal); formal analysis (equal); methodology (equal); writing – original draft (equal); writing – review and editing (equal). **David Bellwood:** Conceptualization (equal); funding acquisition (equal); methodology (equal); supervision (equal); writing – review and editing (equal). **Renato A. Morais:** Conceptualization (equal); data curation (equal); formal analysis (equal); funding acquisition (equal); methodology (equal); supervision (equal); writing – review and editing (equal).

## CONFLICT OF INTEREST

The authors declare no conflicts of interest.

## DATA SOURCES (WHERE APPROPRIATE)

Original data collected, also used in Morais and Bellwood et al. (2019), Morais, Connolly, and Bellwood (2020), Morais, Depczynski, et al. (2020), and Morais et al. (2021). Trait data used in growth and mortality modeling compiled from multiple data sources, available in “Key Resources Table” in Morais and Bellwood (2019) and “Zenodo Repository” in Morais et al. (2021).

### OPEN RESEARCH BADGES

This article has earned Open Data and Open Materials badges. Data and materials are available at https://doi.org/10.25903/devh‐2h34.

## Supporting information


**Appendix S1** Supporting Information.

## Data Availability

Data and code required to reproduce all the analyses and results are made available in “Research Data JCU,” a public data repository hosted by James Cook University (https://doi.org/10.25903/devh‐2h34).
