# Supplementary material for: The role of nocturnal fishes on coral reefs: A quantitative functional evaluation
Source: Ecol Evol. 2022 Aug 26;12(8):e9249. doi: 10.1002/ece3.9249 (PMC9412246; doi:10.1002/ece3.9249)
Supplement: Supplementary file 1 — Appendix S1 Supporting Information. [file ECE3-12-e9249-s001.docx]

Supporting Information

**Appendix S1: Supplemental Methods**

*Data collection*

To compare the relative contributions of diurnal and nocturnal fishes to community level production of biomass, we carried out a high-resolution visual survey dataset conducted at Lizard Island, a mid-shelf reef at the northern portion of the Great Barrier Reef (GBR) (Figure 1), between April 2017 and December 2018. All surveys were conducted by the same experienced observer ([Insert Co-authors name]) to keep any observer bias consistent across all counts (e.g., Thompson and Mapstone 1997; Bernard et al. 2013). The surveys were specifically designed to encompass all fishes present, including nocturnal species, an approach that has previously been used to provide complete fish surveys (Ackerman and Bellwood 2002; Morais and Bellwood 2019). Each survey consisted of four overlapping transects, each focussing on a different set of species from the total fish assemblage. During each transect, the number and size (total length [TL], to the nearest cm for fishes <10cm, in 2 cm bins for fishes <20cm and in 5cm bins for fishes 20cm or larger) of all target fish species within the transect area were recorded. The first transect of the survey was conducted as the transect tape (50m) was being laid. This transect surveyed an area of 50m by 5m and focused on the larger (25cm TL or larger), conspicuous, and fast-moving fishes with little site specificity and, hence, a high chance of leaving the survey area when disturbed by divers. This included relatively large parrotfish, surgeonfish, fusiliers, trevally, rabbitfish, groupers, emperors and snappers. In transect two, the diver would swim back along the transect surveying an area of 30m by 5m. This transect focused on the smaller (<25 cm TL), mobile species that are in most cases more site attached, or tend to be less disturbed, than the fishes of the first phase. This included medium-to-small surgeonfishes, parrotfishes, rabbitfishes, groupers, wrasses, fusiliers, as well as water column-dwelling damselfishes (mostly from the genera *Abudefduf, Amblyglyphidodon* and *Chromis*). After reversing direction again, transect three consisted in surveying the same 30m section as before but over a width of 1m (0.5m either side of the tape). On this transect, the observer looked for smaller, non-cryptic fishes which were vertically positioned within approximately 1m of the benthos. Generally, the counts from this transect consisted of damselfishes, cardinalfishes and a few species of small wrasses. During the fourth and final transect, the diver turned around again, surveying a 30m by 1m area back along the tape. During this phase the tape was coiled up during the count/return swim. The fourth transect focused on surveying holes/caves, gaps between coral colonies and overhangs in the reef looking for cryptic, small, or hidden fishes. This included gobies, blennies, triplefins, dottybacks, and pipefishes, but also fishes from families such as squirrelfishes, soldierfishes and cardinalfishes.

*Estimating standing biomass and productivity from underwater fish surveys*

The first step of the statistical analysis was to collect all the variables needed to estimate weight from length data obtained from the visual surveys. To do this, species-specific length-weight conversion factors “a” and “b” were collected from FishBase (Froese, R. and Pauly, D. 2021). With this information, weight for individuals was calculated using the common length-weight conversion formula (Froese 2006):

$$W=aL^{b}$$

Where W=weight in grams, L = total length in centimetres, and “a” and “b” = species-specific length-weight factors (collected from FishBase). From here, the next step was to calculate the growth of individuals, as well as their instantaneous rate of mortality, the key components to estimate productivity (Morais and Bellwood 2020).

Growth followed the von Bertalanffy Growth Function (VBGF), which requires first predicting the expected growth coefficient at the theoretical maximum size (K_max_) for each of the species surveyed. We used the model from (Morais and Bellwood 2018) to predict K_max_. To do that, we collected a series of species-specific details, as well as the environmental temperature. Environmental temperature consisted in the *in-situ* average long-term sea surface temperature (mean of the multi-year daily average temperatures) at a depth of 2-5m for Lizard Island (from the Lizard Island FAIMMS, IMOS (Australian Institute of Marine Science (AIMS) 2020)). Species-specific details consisted of diet (divided into 7 main categories), vertical position/association with the reef (broken into 6 main categories) and species maximum size (TL in cm) (see Morais and Bellwood 2018 for details on these variables).

Once sea surface temperature, diet, position/association with the reef and maximum species body size were collected, K_max_ was predicted as the median from 1000 bootstrapped Boosted Regression Tree iterations using the “predKmax” function from the “rfishprod” package (Morais and Bellwood 2020) for the software R (R Core Team 2021). Once we had predicted K_max_, it could then be used, in association with species maximum size and individual size as recorded from the survey, to calculate the expected somatic growth of each individual. This was done using the “somaGain” function from the above mentioned “rfishprod” package. This routine estimates the body mass an individual reef fish is expected to gain over the course of a given time period (here set as one day) due to somatic growth (following its expected VBGF trajectory). This is done for each individual fish by first estimating their ‘operational age’ (i.e., age relative to the age of cessation of growth), and then by placing them in their expected growth trajectory. In cases in which field estimated individual sizes were greater than their species reported maximum size, we took the conservative approach of considering that field sizes were an overestimate. To adjust for this, we constrained potential overestimated field sizes to 99% of their species maximum size, which allowed for a minor, often negligible growth. A detailed overview of all steps involved in estimating somatic growth, including the equations, can be found in Morais and Bellwood (2020) and Morais et al. (2020).

Besides somatic growth, calculating productivity also requires considering the likelihood of each individual fish suffering mortality. This relies on estimating the instantaneous rate of mortality for each individual fish, which was calculated using the “predM” function in the “rfishprod” package. We chose the ‘Gislason’ method, which uses the empirical relationship between instantaneous mortality rates and individual-level body size, species maximum size and environmental temperature (Gislason et al. 2010). From the instantaneous rate of mortality, we could then calculate the probability that an individual would suffer mortality on the same one-day time period (i.e., post-survey) as somatic growth was estimated. This was used later on to inform our calculation of stochastic mortality.

One of the final steps in the analysis was to account for the fact that each transect within the survey had a different survey area. This meant that we needed to standardise the outputs from the different transects (e.g., biomass or productivity) relative to the area of that transect. To do this each individual fish was assigned a scaling factor (represented by ‘F’ in the following formula), depending on which phase it was detected. This was calculated by the following formula:

$$F=\frac{1}{\frac{P_{A}}{S_{A}}}$$

Where P_A_ = the area (m^2^) of the transect in which the individual was counted (e.g., transect one had: $50m x 5m=250m^{2}$); S_A_ = the final area (m^2^) for which all transects would be standardised, in this case we used 100m^2^. Multiplying individual body mass or growth by this scaling factor ensured all calculations were standardised per 100m^2^.

Once the body mass and growth of each individual had been standardised by area, the final step was to account for mortality. This was done stochastically over 1,000 iterations and used the rfishprod “applyMstoch” function. The applyMstoch function uses a Bernoulli distribution to assign fate (i.e., survival or mortality) to individual fishes based on their instantaneous mortality probability, as calculated in previous steps. In practical terms, at each iteration and for each individual, it outputs True (survival) or False (mortality). Individuals assigned mortality were then removed from the dataset so that their growth would not contribute to the productivity at the next time (i.e., one day after the survey). These individuals still contributed to the aggregate biomass at the time of the survey. This procedure of stochastic assignment of mortality was repeated for 1,000 iterations to simulate average mortality rates, with the somatic growth of surviving fishes averaged across all iterations. Biomass and productivity were calculated for each sample (survey) by summing individual body masses and average productivities of all fishes in that survey. Finally, biomass and productivity were averaged across samples for sheltered and exposed locations, and for nocturnal and diurnal representatives of each family.

*Data analysis*

In order to test for differences in reef fish productivity and biomass between site types and diel habits (nocturnal vs diurnal), we used two Bayesian generalised linear mixed effect models with Gamma distribution and log link function. Each model included either biomass or productivity as the response variable. For each model, both habits and site type were used as explanatory variables, and survey number (sample) nested within site was included as a random effect. We used the No-U-Turn Markov Chain Monte Carlo (MCMC) sampler in Stan via ‘rstan’ (Stan Development Team 2020) and ‘rstanarm’ (Goodrich et al. 2020) in R. For each model, we used 5,000 iterations per chain in a total of four chains, including a 50% burn-in. We specified normally distributed priors (mean = 0, SD = 10) for both intercept and coefficients (habits and site types), and an exponentially distributed auxiliary prior (rate = 1). Each model was examined with trace plots (function ‘mcmc_trace’), R-hat diagnostics (function ‘mcmc_rhat_hist’), autocorrelation function (function ‘mcmc_acf’) and posterior predictive checks (function ‘pp_check’). This ensured that the models were correctly specified, converged, showed low autocorrelation between MCMC steps, and were adequately modelling the data. We then calculated marginal means, lower/upper 95% High Posterior Density intervals (HPD) for each combination of site type and habit (parameter) from each model using the ‘emmeans’ (Lenth 2021) package. Contrasts were generated by the ratios between each two intended coefficients (e.g., nocturnal fish biomass divided by diurnal fish biomass), and were obtained by specifying ‘pairwise’ comparisons within emmeans at the response scale. Examining the HPDs of the ratios between combination of site type and habit then allowed me to design significance tests (see below formula).

$$Lower.HPD\leq1\leq Upper.HPD=Non significant$$

*NMDS*

To inspect the taxonomic (family) composition of the productivity and biomass of nocturnal fishes and how they varied between exposed and sheltered site types, we used non-metric multidimensional scaling (NMDS). These were performed using the ‘metaMDS’ function from the ‘vegan’ package in R, which first applies square-root and double Wisconsin standardisation, and then automatically calculates the Bray-Curtis dissimilarity between pairs of samples. We set the number of dimensions to two. We plotted the two-dimensions of the resulting NMDS, including family vectors and the stress, a metric of how correlated the NMDS points are with the real dissimilarity data.

To identify the most important families of nocturnal reef fishes, between site types, we first visually examined their family-level biomass and productivity. This was done through inspecting sample values and their 50% and 95% quantiles. This suggested that apogonids were the highest-ranking family in terms of their combined contribution to biomass and productivity, also featuring the largest differences between sheltered and exposed sites. To further explore the differences in Apogonidae biomass and productivity between site types, we again used two Bayesian generalised linear mixed effect models, both with a Gamma distribution and log link function. Each model included either biomass or productivity as the response variable. For each model, site type was used as an explanatory variable, and site was included as a random effect. The same MCMC sampler as above was used. For each model, we used 5,000 iterations per chain in a total of four chains, including a 50% burn-in. Priors were not specified meaning that both models used rstanarm’s default priors. These consisted of normally distributed priors for both the intercept (mean = 0, SD = 2.5) and the coefficient (mean = 0, SD = 5.4), and an exponentially distributed auxiliary prior (rate = 1). Each model was examined with the same trace plots, R-hat diagnostics, autocorrelation function and posterior predictive checks as before. Marginal means, HPDs and contrasts were calculated as before to test for pairwise differences.

**Supplemental References**

Ackerman JL, Bellwood DR (2002) Comparative efficiency of clove oil and rotenone for sampling tropical reef fish assemblages. J Fish Biol 60:893–901

Australian Institute of Marine Science (AIMS) (2020) Northern Australia Automated Marine Weather and Oceanographic Stations, Sites: [Lizard Island]. https://doi.org/10.25845/5c09bf93f315d, accessed 28-Jul-2021

Basford AJ, Feary DA, Truong G, Steinberg PD, Marzinelli EM, Vergés A, Basford AJ, Feary DA, Truong G, Steinberg PD, Marzinelli EM, Vergés A (2016) Feeding habits of range-shifting herbivores: tropical surgeonfishes in a temperate environment. Mar Freshw Res 67:75–83

Bellwood DR, Hughes TP, Hoey AS (2006) Sleeping Functional Group Drives Coral-Reef Recovery. Curr Biol 16:2434–2439

Bernard ATF, Götz A, Kerwath SE, Wilke CG (2013) Observer bias and detection probability in underwater visual census of fish assemblages measured with independent double-observers. J Exp Mar Biol Ecol 443:75–84

Boaden AE, Kingsford MJ (2012) Diel behaviour and trophic ecology of Scolopsis bilineatus (Nemipteridae). Coral Reefs 31:871–883

Bosiger YJ, McCormick MI (2014) Temporal Links in Daily Activity Patterns between Coral Reef Predators and Their Prey. PLOS ONE 9:e111723

Brandl SJ, Bellwood DR (2013) Pair formation in the herbivorous rabbitfish Siganus doliatus. J Fish Biol 82:2031–2044

Bshary R, Hohner A, Ait-el-Djoudi K, Fricke H (2006) Interspecific Communicative and Coordinated Hunting between Groupers and Giant Moray Eels in the Red Sea. PLOS Biol 4:e431

Chase TJ, Nowicki JP, Coker DJ (2018) Diurnal foraging of a wild coral-reef fish Parapercis australis in relation to late-summer temperatures. J Fish Biol 93:153–158

Chen T-C, Ormond RFG, Mok H-K (2001) Feeding and territorial behaviour in juveniles of three co-existing triggerfishes. J Fish Biol 59:524–532

Daly R, Filmalter J, Daly C, Bennett R, Pereira M, Mann B, Dunlop S, Cowley P (2019) Acoustic telemetry reveals multi-seasonal spatiotemporal dynamics of a giant trevally Caranx ignobilis aggregation. Mar Ecol Prog Ser 621:185–197

Fishelson L, Popper D, Gunderman N (1971) Diurnal cyclic behaviour of Pempheris oualensis Cuv. & Val. (Pempheridae, Teleostei). J Nat Hist 5:503–506

Fitzpatrick C, McLean D, Harvey ES (2013) Using artificial illumination to survey nocturnal reef fish. Fish Res 146:41–50

Fox RJ, Bellwood DR (2011) Unconstrained by the clock? Plasticity of diel activity rhythm in a tropical reef fish, Siganus lineatus: Diel activity plasticity in a rabbitfish. Funct Ecol 25:1096–1105

Froese, R., Pauly, D. (2021) Search FishBase. https://www.fishbase.se/search.php

Gislason H, Daan N, Rice JC, Pope JG (2010) Size, growth, temperature and the natural mortality of marine fish. Fish Fish 11:149–158

Goodrich B, Gabry J, Ali I, Brilleman S (2020) rstanarm: Bayesian applied regression modeling via Stan.

Haight WR, Parrish JD, Hayes TA (2011) Feeding Ecology of Deepwater Lutjanid Snappers at Penguin Bank, Hawaii. Trans Am Fish Soc 122:328–347

Hamilton RJ, Giningele M, Aswani S, Ecochard JL (2012) Fishing in the dark-local knowledge, night spearfishing and spawning aggregations in the Western Solomon Islands. Biol Conserv 145:246–257

Harmelin-Vivien, Bouchon (1976) Feeding Behavior of Some Carnivorous Fishes (Serranidae and Scorpaenidae) from Tulear (Madagascar). Mar Biol 37:329–340

Heupel MR, Bennett MB (1998) Observations on the diet and feeding habits of the epaulette shark, Hemiscyllium ocellatum (Bonnaterre), on Heron Island Reef, Great Barrier Reef, Australia. Mar Freshw Res 49:753–756

Holland KN, Peterson JD, Lowe CG, Wetherbee BM (1993) Movements, Distribution and Growth Rates of the White Goatfish Mulloides Flavolineatus in a Fisheries Conservation Zone. Bull Mar Sci 52:982–992

Kawabata Y, Okuyama J, Mitamura H, Asami K, Yoseda K, Arai N (2007) Post-release movement and diel activity patterns of hatchery-reared and wild black-spot tuskfish Choerodon schoenleinii determined by ultrasonic telemetry. Fish Sci 73:1147–1154

Khan JA, Goatley CHR, Brandl SJ, Tebbett SB, Bellwood DR (2017) Shelter use by large reef fishes: long-term occupancy and the impacts of disturbance. Coral Reefs 36:1123–1132

Lenth RV (2021) emmeans: Estimated Marginal Means, aka Least-Squares Means.

Liu M, Sadovy Y (2005) Habitat Association and Social Structure of the Chocolate Hind, Cephalopholis boenak (Pisces: Serranidae: Epinephelinae), at Ping Chau Island, Northeastern Hong Kong Waters. Environ Biol Fishes 74:9–18

Luehrmann M, Cortesi F, Cheney KL, Busserolles F de, Marshall NJ (2020) Microhabitat partitioning correlates with opsin gene expression in coral reef cardinalfishes (Apogonidae). Funct Ecol 34:1041–1052

Marnane M, Bellwood D (2002) Diet and nocturnal foraging in cardinalfishes (Apogonidae) at One Tree Reef, Great Barrier Reef, Australia. Mar Ecol Prog Ser 231:261–268

Meyer CG, Holland KN (2005) Movement patterns, home range size and habitat utilization of the bluespine unicornfish, Naso unicornis (Acanthuridae) in a Hawaiian marine reserve. Environ Biol Fishes 73:201–210

Meyer CG, Holland KN, Papastamatiou YP (2007) Seasonal and diel movements of giant trevally Caranx ignobilis at remote Hawaiian atolls: implications for the design of Marine Protected Areas. Mar Ecol Prog Ser 333:13–25

Morais RA, Bellwood DR (2018) Global drivers of reef fish growth. Fish Fish 19:874–889

Morais RA, Bellwood DR (2019) Pelagic Subsidies Underpin Fish Productivity on a Degraded Coral Reef. Curr Biol 29:1521–1527

Morais RA, Bellwood DR (2020) Principles for estimating fish productivity on coral reefs. Coral Reefs 39:1221–1231

Myers RF (1999) Micronesia Reef Fishes: A Comprehensive Guide to the Coral Reef Fishes of Micronesia, 3rd revised and expanded edition. Coral Graphics, Guam

Nakamura Y, Tsuchiya M (2008) Spatial and temporal patterns of seagrass habitat use by fishes at the Ryukyu Islands, Japan. Estuar Coast Shelf Sci 76:345–356

Nixon AJ, Gruber SH (1988) Diel metabolic and activity patterns of the lemon shark (Negaprion brevirostris). J Exp Zool 248:1–6

Popper D, Gunderman N (1975) Some ecological and behavioural aspects of siganid populations in the Red Sea and Mediterranean coasts of Israel in relation to their suitability for aquaculture. Aquaculture 6:127–141

R Core Team (2021) R: A language and environment for statistical computing. R Foundation for Statistical Computing, Vienna, Austria

Randall JE, Allen GR, Steene RC (1998) Fishes of the Great Barrier Reef and Coral Sea. Crawford House Publishing, Bathurst, Australia

Shpigel M, Fishelson L (1989a) Habitat partitioning between species of the genus Cephalopholis (Pisces, Serranidae) across the fringing reef of the Gulf of Aqaba (Red Sea). Mar Ecol Prog Ser 58:17–22

Shpigel M, Fishelson L (1989b) Food habits and prey selection of three species of groupers from the genus Cephalopholis (Serranidae: Teleostei). Environ Biol Fishes 24:67–73

Stan Development Team (2020) RStan: the R interface to Stan.

Sylva DPD (1973) Barracudas (Pisces: Sphyraenidae) of the Ocean and Adjacent Seas - A Preliminary Review of their Systematics and Ecology. J Mar Biol Assoc India 15:74–94

Takegaki T (2001) Environmental factors affecting the spawning burrow selection by the gobiid Valenciennea longipinnis. J Fish Biol 58:222–229

Thompson A, Mapstone B (1997) Observer effects and training in underwater visual surveys of reef fishes. Mar Ecol Prog Ser 154:53–63

Whitney N, Papastamatiou Y, Holland K, Lowe C (2007) Use of an acceleration data logger to measure diel activity patterns in captive whitetip reef sharks, Triaenodon obesus. Aquat Living Resour 20:299–305

Zeller D (1997) Home range and activity patterns of the coral trout Plectropomus leopardus (Serranidae). Mar Ecol Prog Ser 154:65–77


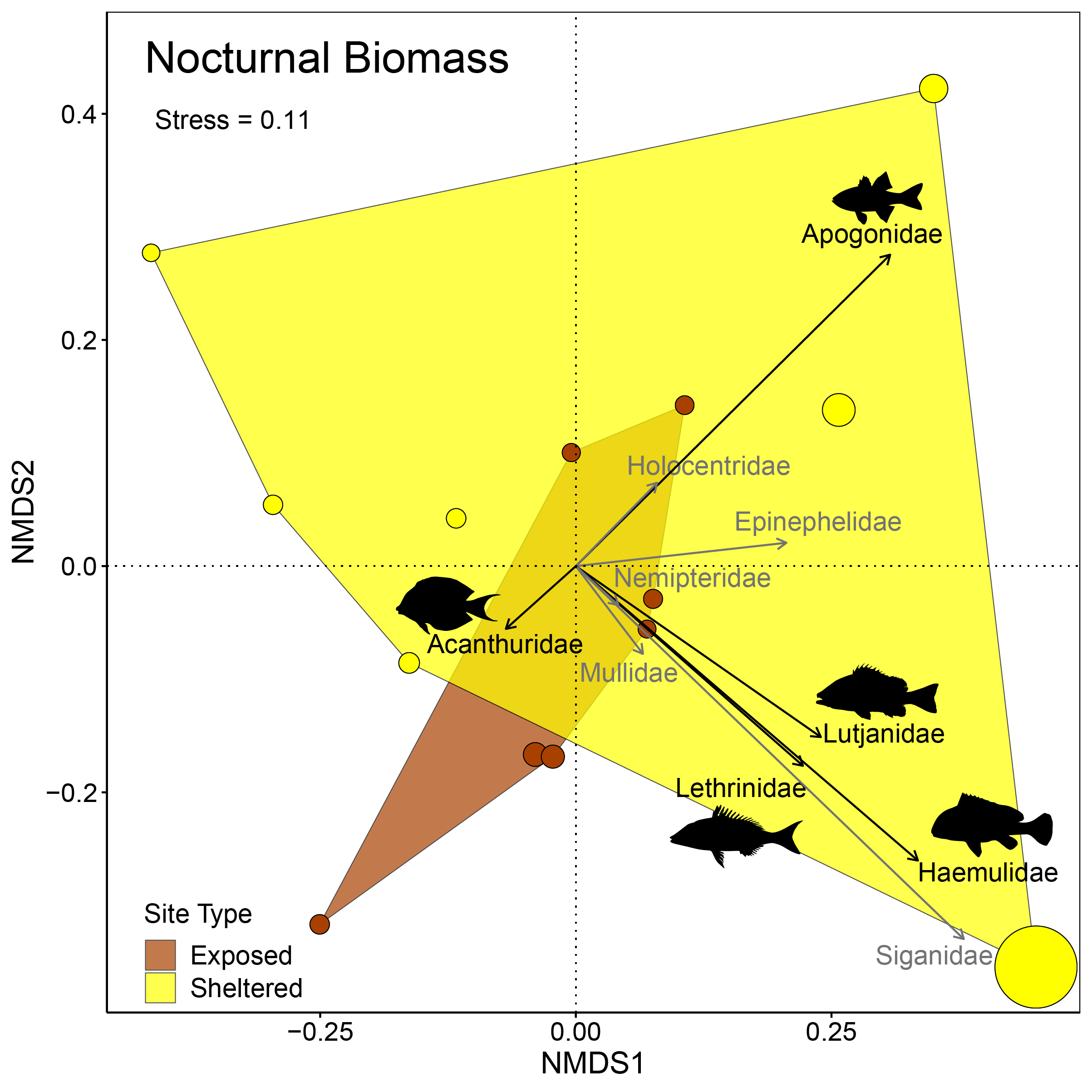


Figure S1: Site level NMDS plots of (exposed in brown, sheltered in yellow) biomass for the top 10 nocturnal fish families (based on their contributions to productivity). Top 5 families are in represented by the colour black and the top 6-10 families are in grey. Dot size represents the relative biomass of each site (diameter scaled to productivity).

**
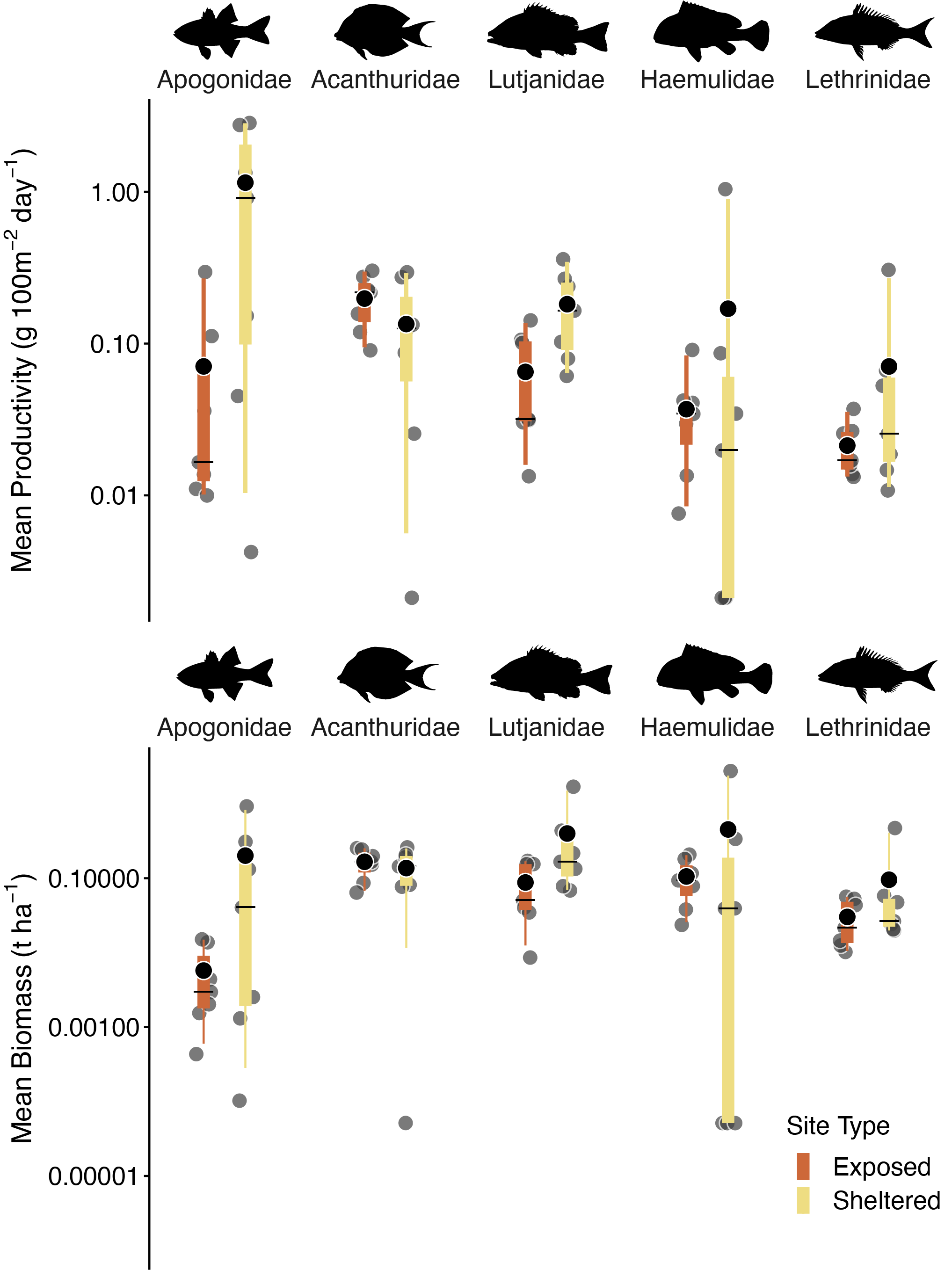
**

Figure S2: Biomass and productivity (log scale) of the 5 top-ranking nocturnal fish families (ordered by productivity). Grey dots represent the raw data. Solid black dots represent the mean and thin lines represent the median. The thick bars represent the 50% confidence interval, and the thin bars represent the 95 percent confidence interval.

Table S1: Species list used to define activity type of different fish species

| **Family** | **Species** | **Activity Type** | **Reason** | **Reference** |
| --- | --- | --- | --- | --- |
| Acanthuridae | Acanthurus auranticavus | Diurnal | Observations | field observations |
| Acanthuridae | Acanthurus blochii | Diurnal | Observations | field observations |
| Acanthuridae | Acanthurus dussumieri | Both | Reference | (Basford et al. 2016; Khan et al. 2017) |
| Acanthuridae | Acanthurus lineatus | Diurnal | Observations | field observations |
| Acanthuridae | Acanthurus nigricauda | Diurnal | Observations | field observations |
| Acanthuridae | Acanthurus nigrofuscus | Diurnal | Observations | field observations |
| Acanthuridae | Acanthurus olivaceus | Diurnal | Observations | field observations |
| Acanthuridae | Acanthurus pyroferus | Diurnal | Observations | field observations |
| Acanthuridae | Acanthurus xanthopterus | Diurnal | Observations | field observations |
| Acanthuridae | Ctenochaetus binotatus | Diurnal | Observations | field observations |
| Acanthuridae | Ctenochaetus sp | Diurnal | Observations | field observations |
| Acanthuridae | Ctenochaetus striatus | Diurnal | Observations | field observations |
| Acanthuridae | Naso annulatus | Diurnal | Observations | field observations |
| Acanthuridae | Naso brachycentron | Diurnal | Observations | field observations |
| Acanthuridae | Naso brevirostris | Diurnal | Observations | field observations |
| Acanthuridae | Naso hexacanthus | Diurnal | Observations | field observations |
| Acanthuridae | Naso lituratus | Diurnal | Observations | field observations |
| Acanthuridae | Naso tonganus | Diurnal | Observations | field observations |
| Acanthuridae | Naso unicornis | Both | Reference | (Meyer and Holland 2005) |
| Acanthuridae | Naso vlamingii | Diurnal | Observations | field observations |
| Acanthuridae | Zebrasoma scopas | Diurnal | Observations | field observations |
| Acanthuridae | Zebrasoma velifer | Diurnal | Observations | field observations |
| Apogonidae | Cheilodipterus artus | Nocturnal | Reference | (Marnane and Bellwood 2002) |
| Apogonidae | Cheilodipterus intermedius | Nocturnal | Inference | Phylogeny |
| Apogonidae | Cheilodipterus macrodon | Nocturnal | Inference | Phylogeny |
| Apogonidae | Cheilodipterus quinquelineatus | Nocturnal | Reference | (Marnane and Bellwood 2002; Nakamura and Tsuchiya 2008) |
| Apogonidae | Fibramia thermalis | Nocturnal | Inference | Phylogeny |
| Apogonidae | Ostorhinchus compressus | Nocturnal | Inference | Phylogeny |
| Apogonidae | Ostorhinchus cookii | Nocturnal | Inference | Phylogeny |
| Apogonidae | Ostorhinchus cyanosoma | Nocturnal | Reference | (Marnane and Bellwood 2002) |
| Apogonidae | Ostorhinchus doederleini | Nocturnal | Reference | (Marnane and Bellwood 2002; Fitzpatrick et al. 2013) |
| Apogonidae | Ostorhinchus neotes | Nocturnal | Inference | Phylogeny |
| Apogonidae | Ostorhinchus nigrofasciatus | Nocturnal | Inference | Phylogeny |
| Apogonidae | Ostorhinchus novemfasciatus | Nocturnal | Inference | Phylogeny |
| Apogonidae | Pristiapogon fraenatus | Nocturnal | Inference | Phylogeny |
| Apogonidae | Rhabdamia gracilis | Diurnal | Reference | (Luehrmann et al. 2020) |
| Apogonidae | Taeniamia fucata | Nocturnal | Inference | Phylogeny |
| Apogonidae | Taeniamia zosterophora | Nocturnal | Inference | Phylogeny |
| Apogonidae | Verulux cypselurus | Nocturnal | Inference | Phylogeny |
| Apogonidae | Zoramia leptacantha | Nocturnal | Inference | Phylogeny |
| Apogonidae | Zoramia viridiventer | Nocturnal | Inference | Phylogeny |
| Balistidae | Balistapus undulatus | Diurnal | Reference | (Froese, R. and Pauly, D. 2021) |
| Balistidae | Balistoides viridescens | Diurnal | Reference | (Chen et al. 2001) |
| Balistidae | Pseudobalistes flavimarginatus | Diurnal | Observations | field observations |
| Balistidae | Rhinecanthus aculeatus | Diurnal | Observations | field observations |
| Balistidae | Sufflamen chrysopterum | Diurnal | Observations | field observations |
| Blenniidae | Atrosalarias fuscus | Diurnal | Observations | field observations |
| Blenniidae | Blenniella chrysospilos | Diurnal | Observations | field observations |
| Blenniidae | Cirripectes filamentosus | Diurnal | Observations | field observations |
| Blenniidae | Cirripectes sp | Diurnal | Observations | field observations |
| Blenniidae | Crossosalarias macrospilus | Diurnal | Observations | field observations |
| Blenniidae | Ecsenius bicolor | Diurnal | Observations | field observations |
| Blenniidae | Ecsenius sp | Diurnal | Observations | field observations |
| Blenniidae | Ecsenius stictus | Diurnal | Observations | field observations |
| Blenniidae | Exallias brevis | Diurnal | Observations | field observations |
| Blenniidae | Glyptoparus delicatulus | Diurnal | Observations | field observations |
| Blenniidae | Meiacanthus atrodorsalis | Diurnal | Observations | field observations |
| Blenniidae | Meiacanthus grammistes | Diurnal | Observations | field observations |
| Blenniidae | Plagiotremus rhinorhynchos | Diurnal | Observations | field observations |
| Blenniidae | Plagiotremus tapeinosoma | Diurnal | Observations | field observations |
| Blenniidae | Salarias alboguttatus | Diurnal | Observations | field observations |
| Blenniidae | Salarias fasciatus | Diurnal | Observations | field observations |
| Blenniidae | Salarias guttatus | Diurnal | Observations | field observations |
| Blenniidae | Salarias sp | Diurnal | Observations | field observations |
| Callionymidae | Diplogrammus goramensis | Diurnal | Observations | field observations |
| Carangidae | Caranx ignobilis | Both | Reference | (Meyer et al. 2007; Daly et al. 2019) |
| Carangidae | Caranx melampygus | Diurnal | Observations | field observations |
| Carangidae | Caranx papuensis | Diurnal | Observations | field observations |
| Carangidae | Caranx sexfasciatus | Diurnal | Observations | field observations |
| Carangidae | Scomberoides lysan | Diurnal | Observations | field observations |
| Carcharhinidae | Carcharhinus melanopterus | Nocturnal | Observations | Relatives & field observations |
| Carcharhinidae | Negaprion brevirostris | Nocturnal | Reference | (Nixon and Gruber 1988) |
| Carcharhinidae | Triaenodon obesus | Nocturnal | Reference | (Whitney et al. 2007) |
| Chaetodontidae | Chaetodon aureofasciatus | Diurnal | Observations | field observations |
| Chaetodontidae | Chaetodon auriga | Diurnal | Observations | field observations |
| Chaetodontidae | Chaetodon baronessa | Diurnal | Observations | field observations |
| Chaetodontidae | Chaetodon citrinellus | Diurnal | Observations | field observations |
| Chaetodontidae | Chaetodon ephippium | Diurnal | Observations | field observations |
| Chaetodontidae | Chaetodon flavirostris | Diurnal | Observations | field observations |
| Chaetodontidae | Chaetodon kleinii | Diurnal | Observations | field observations |
| Chaetodontidae | Chaetodon lineolatus | Diurnal | Observations | field observations |
| Chaetodontidae | Chaetodon lunula | Diurnal | Observations | field observations |
| Chaetodontidae | Chaetodon lunulatus | Diurnal | Observations | field observations |
| Chaetodontidae | Chaetodon melannotus | Diurnal | Observations | field observations |
| Chaetodontidae | Chaetodon plebeius | Diurnal | Observations | field observations |
| Chaetodontidae | Chaetodon rafflesii | Diurnal | Observations | field observations |
| Chaetodontidae | Chaetodon rainfordi | Diurnal | Observations | field observations |
| Chaetodontidae | Chaetodon trifascialis | Diurnal | Observations | field observations |
| Chaetodontidae | Chaetodon ulietensis | Diurnal | Observations | field observations |
| Chaetodontidae | Chaetodon unimaculatus | Diurnal | Observations | field observations |
| Chaetodontidae | Chaetodon vagabundus | Diurnal | Observations | field observations |
| Chaetodontidae | Chelmon rostratus | Diurnal | Observations | field observations |
| Chaetodontidae | Heniochus acuminatus | Diurnal | Observations | field observations |
| Chaetodontidae | Heniochus chrysostomus | Nocturnal | Observations | field observations |
| Chaetodontidae | Heniochus monoceros | Nocturnal | Observations | field observations |
| Chaetodontidae | Heniochus singularius | Nocturnal | Observations | field observations |
| Chaetodontidae | Heniochus varius | Nocturnal | Observations | field observations |
| Cirrhitidae | Cirrhitichthys falco | Diurnal | Observations | field observations |
| Cirrhitidae | Cirrhitichthys oxycephalus | Diurnal | Observations | field observations |
| Cirrhitidae | Paracirrhites forsteri | Diurnal | Observations | field observations |
| Clupeidae | Spratelloides spp | Diurnal | Observations | field observations |
| Dasyatidae | Himantura australis | Both | Observations | field observations |
| Dasyatidae | Neotrygon kuhlii | Both | Observations | field observations |
| Dasyatidae | Taeniura lymma | Both | Observations | field observations |
| Echeneidae | Echeneis naucrates | Diurnal | Observations | field observations |
| Ephippidae | Platax orbicularis | Diurnal | Observations | field observations |
| Ephippidae | Platax pinnatus | Diurnal | Reference | (Bellwood et al. 2006b) |
| Ephippidae | Platax teira | Diurnal | Observations | field observations |
| Epinephelidae | Cephalopholis argus | Both | Reference | (Harmelin-Vivien and Bouchon 1976; Shpigel and Fishelson 1989a, 1989b) |
| Epinephelidae | Cephalopholis boenak | Diurnal | Reference | (Liu and Sadovy 2005) |
| Epinephelidae | Cephalopholis cyanostigma | Both | Reference | (Bosiger and McCormick 2014) |
| Epinephelidae | Cephalopholis microprion | Diurnal | Observations | field observations |
| Epinephelidae | Cephalopholis sp | Diurnal | Observations | field observations |
| Epinephelidae | Cephalopholis urodeta | Diurnal | Observations | field observations |
| Epinephelidae | Cromileptes altivelis | Diurnal | Observations | field observations |
| Epinephelidae | Epinephelus fasciatus | Both | Reference | (Harmelin-Vivien and Bouchon 1976) |
| Epinephelidae | Epinephelus maculatus | Diurnal | Assumed | field observations |
| Epinephelidae | Epinephelus merra | Both | Reference | (Harmelin-Vivien and Bouchon 1976) |
| Epinephelidae | Plectropomus areolatus | Both | Reference | (Hamilton et al. 2012) |
| Epinephelidae | Plectropomus laevis | Diurnal | Assumed | field observations |
| Epinephelidae | Plectropomus leopardus | Diurnal | Reference | (Zeller 1997) |
| Fistulariidae | Fistularia commersonii | Diurnal | Observations | field observations |
| Ginglymostomatidae | Nebrius ferrugineus | Nocturnal | Observations | field observations |
| Gobiidae | Aioliops tetrophthalmus | Diurnal | Observations | field observations |
| Gobiidae | Amblyeleotris guttata | Diurnal | Observations | field observations |
| Gobiidae | Amblyeleotris steinitzi | Diurnal | Observations | field observations |
| Gobiidae | Amblyeleotris wheeleri | Diurnal | Observations | field observations |
| Gobiidae | Amblygobius decussatus | Diurnal | Observations | field observations |
| Gobiidae | Amblygobius nocturnus | Diurnal | Observations | field observations |
| Gobiidae | Amblygobius phalaena | Diurnal | Observations | field observations |
| Gobiidae | Asterropteryx semipunctata | Diurnal | Observations | field observations |
| Gobiidae | Bryaninops sp | Diurnal | Observations | field observations |
| Gobiidae | Cryptocentrus strigilliceps | Diurnal | Observations | field observations |
| Gobiidae | Ctenogobiops mitodes | Diurnal | Observations | field observations |
| Gobiidae | Ctenogobiops pomastictus | Diurnal | Observations | field observations |
| Gobiidae | Ctenogobiops sp | Diurnal | Observations | field observations |
| Gobiidae | Eviota atriventris | Diurnal | Observations | field observations |
| Gobiidae | Eviota melasma | Diurnal | Observations | field observations |
| Gobiidae | Eviota prasites | Diurnal | Observations | field observations |
| Gobiidae | Eviota queenslandica | Diurnal | Observations | field observations |
| Gobiidae | Eviota sebreei | Diurnal | Observations | field observations |
| Gobiidae | Eviota sigillata | Diurnal | Observations | field observations |
| Gobiidae | Eviota sp | Diurnal | Observations | field observations |
| Gobiidae | Eviota teresae | Diurnal | Observations | field observations |
| Gobiidae | Eviota zebrina | Diurnal | Observations | field observations |
| Gobiidae | Fusigobius duospilus | Diurnal | Observations | field observations |
| Gobiidae | Fusigobius humeralis | Diurnal | Observations | field observations |
| Gobiidae | Fusigobius melacron | Diurnal | Observations | field observations |
| Gobiidae | Fusigobius neophytus | Diurnal | Observations | field observations |
| Gobiidae | Fusigobius signipinnis | Diurnal | Observations | field observations |
| Gobiidae | Fusigobius sp | Diurnal | Observations | field observations |
| Gobiidae | Fusigobius spp | Diurnal | Observations | field observations |
| Gobiidae | Gnatholepis anjerensis | Diurnal | Observations | field observations |
| Gobiidae | Gnatholepis cauerensis | Diurnal | Observations | field observations |
| Gobiidae | Gobiodon aoyagii | Diurnal | Observations | field observations |
| Gobiidae | Gobiodon histrio | Diurnal | Observations | field observations |
| Gobiidae | Gobiodon quinquestrigatus | Diurnal | Observations | field observations |
| Gobiidae | Gobiodon rivulatus | Diurnal | Observations | field observations |
| Gobiidae | Gobiodon sp | Diurnal | Observations | field observations |
| Gobiidae | Istigobius decoratus | Diurnal | Observations | field observations |
| Gobiidae | Istigobius goldmanni | Diurnal | Observations | field observations |
| Gobiidae | Istigobius rigilius | Diurnal | Observations | field observations |
| Gobiidae | Istigobius sp | Diurnal | Observations | field observations |
| Gobiidae | Koumansetta rainfordi | Diurnal | Observations | field observations |
| Gobiidae | Pleurosicya micheli | Diurnal | Observations | field observations |
| Gobiidae | Pleurosicya sp | Diurnal | Observations | field observations |
| Gobiidae | Ptereleotris evides | Diurnal | Observations | field observations |
| Gobiidae | Ptereleotris heteroptera | Diurnal | Observations | field observations |
| Gobiidae | Ptereleotris microlepis | Diurnal | Observations | Field observations |
| Gobiidae | Ptereleotris zebra | Diurnal | Observations | Field observations |
| Gobiidae | Signigobius biocellatus | Diurnal | Observations | Field observations |
| Gobiidae | Trimma lantana | Diurnal | Observations | Field observations |
| Gobiidae | Trimma sp | Diurnal | Observations | Field observations |
| Gobiidae | Trimma striatum | Diurnal | Observations | Field observations |
| Gobiidae | Valenciennea longipinnis | Diurnal | Reference | (Takegaki 2001) |
| Gobiidae | Valenciennea parva | Diurnal | Observations | Field observations |
| Gobiidae | Valenciennea puellaris | Diurnal | Observations | Field observations |
| Gobiidae | Valenciennea strigata | Diurnal | Observations | Field observations |
| Haemulidae | Diagramma pictum | Both | Observations | Field observations |
| Haemulidae | Plectorhinchus albovittatus | Nocturnal | Observations | Field observations |
| Haemulidae | Plectorhinchus chaetodonoides | Nocturnal | Reference | (Myers 1999; Khan et al. 2017) |
| Haemulidae | Plectorhinchus chrysotaenia | Nocturnal | Observations | Field observations |
| Haemulidae | Plectorhinchus flavomaculatus | Nocturnal | Observations | Field observations |
| Haemulidae | Plectorhinchus gibbosus | Nocturnal | Reference | (Khan et al. 2017) |
| Haemulidae | Plectorhinchus lineatus | Nocturnal | Reference | (Myers 1999; Khan et al. 2017) |
| Hemiscylliidae | Hemiscyllium ocellatum | Both | Reference | (Heupel and Bennett 1998) |
| Holocentridae | Myripristis berndti | Nocturnal | Reference | (Randall et al. 1998; Myers 1999) |
| Holocentridae | Myripristis kuntee | Nocturnal | Reference | (Randall et al. 1998; Myers 1999) |
| Holocentridae | Myripristis violacea | Nocturnal | Reference | (Randall et al. 1998; Myers 1999) |
| Holocentridae | Neoniphon sammara | Nocturnal | Reference | (Randall et al. 1998; Myers 1999) |
| Holocentridae | Sargocentron cornutum | Nocturnal | Reference | (Randall et al. 1998; Myers 1999) |
| Holocentridae | Sargocentron spiniferum | Nocturnal | Reference | (Randall et al. 1998; Myers 1999) |
| Kyphosidae | Kyphosus cinerascens | Diurnal | Observations | Field observations |
| Kyphosidae | Kyphosus vaigiensis | Diurnal | Observations | Field observations |
| Labridae | Anampses caeruleopunctatus | Diurnal | Observations | Field observations |
| Labridae | Anampses geographicus | Diurnal | Observations | Field observations |
| Labridae | Anampses meleagrides | Diurnal | Observations | Field observations |
| Labridae | Anampses neoguinaicus | Diurnal | Observations | Field observations |
| Labridae | Bodianus axillaris | Diurnal | Observations | Field observations |
| Labridae | Bodianus dictynna | Diurnal | Observations | Field observations |
| Labridae | Bodianus mesothorax | Diurnal | Observations | Field observations |
| Labridae | Bolbometopon muricatum | Diurnal | Observations | Field observations |
| Labridae | Cetoscarus ocellatus | Diurnal | Observations | Field observations |
| Labridae | Cheilinus chlorourus | Diurnal | Observations | Field observations |
| Labridae | Cheilinus fasciatus | Diurnal | Observations | Field observations |
| Labridae | Cheilinus oxycephalus | Diurnal | Observations | Field observations |
| Labridae | Cheilinus trilobatus | Diurnal | Observations | Field observations |
| Labridae | Cheilinus undulatus | Diurnal | Observations | Field observations |
| Labridae | Chlorurus bleekeri | Diurnal | Observations | Field observations |
| Labridae | Chlorurus microrhinos | Diurnal | Observations | Field observations |
| Labridae | Chlorurus spilurus | Diurnal | Observations | Field observations |
| Labridae | Choerodon anchorago | Diurnal | Observations | Field observations |
| Labridae | Choerodon fasciatus | Diurnal | Observations | Field observations |
| Labridae | Choerodon schoenleinii | Diurnal | Reference | (Kawabata et al. 2007) |
| Labridae | Cirrhilabrus exquisitus | Diurnal | Observations | Field observations |
| Labridae | Coris aygula | Diurnal | Observations | Field observations |
| Labridae | Coris batuensis | Diurnal | Observations | Field observations |
| Labridae | Coris dorsomacula | Diurnal | Observations | Field observations |
| Labridae | Coris gaimard | Diurnal | Observations | Field observations |
| Labridae | Epibulus brevis | Diurnal | Observations | Field observations |
| Labridae | Epibulus insidiator | Diurnal | Observations | Field observations |
| Labridae | Gomphosus varius | Diurnal | Observations | Field observations |
| Labridae | Halichoeres chloropterus | Diurnal | Observations | Field observations |
| Labridae | Halichoeres hortulanus | Diurnal | Observations | Field observations |
| Labridae | Halichoeres marginatus | Diurnal | Observations | Field observations |
| Labridae | Halichoeres melanurus | Diurnal | Observations | Field observations |
| Labridae | Halichoeres nebulosus | Diurnal | Observations | Field observations |
| Labridae | Halichoeres prosopeion | Diurnal | Observations | Field observations |
| Labridae | Halichoeres sp | Diurnal | Observations | Field observations |
| Labridae | Halichoeres trimaculatus | Diurnal | Reference | (Nakamura and Tsuchiya 2008) |
| Labridae | Hemigymnus fasciatus | Diurnal | Observations | Field observations |
| Labridae | Hemigymnus melapterus | Diurnal | Observations | Field observations |
| Labridae | Hipposcarus longiceps | Diurnal | Observations | Field observations |
| Labridae | Hologymnosus annulatus | Diurnal | Observations | Field observations |
| Labridae | Hologymnosus doliatus | Diurnal | Observations | Field observations |
| Labridae | Labrichthys unilineatus | Diurnal | Observations | Field observations |
| Labridae | Labroides bicolor | Diurnal | Observations | Field observations |
| Labridae | Labroides dimidiatus | Diurnal | Observations | Field observations |
| Labridae | Macropharyngodon meleagris | Diurnal | Observations | Field observations |
| Labridae | Macropharyngodon negrosensis | Diurnal | Observations | Field observations |
| Labridae | Novaculichthys taeniourus | Diurnal | Observations | Field observations |
| Labridae | Oxycheilinus digramma | Diurnal | Observations | Field observations |
| Labridae | Oxycheilinus orientalis | Diurnal | Observations | Field observations |
| Labridae | Oxycheilinus sp | Diurnal | Observations | Field observations |
| Labridae | Pseudocheilinus evanidus | Diurnal | Observations | Field observations |
| Labridae | Pseudocheilinus hexataenia | Diurnal | Observations | Field observations |
| Labridae | Pteragogus cryptus | Diurnal | Observations | Field observations |
| Labridae | Scarus altipinnis | Diurnal | Observations | Field observations |
| Labridae | Scarus chameleon | Diurnal | Observations | Field observations |
| Labridae | Scarus dimidiatus | Diurnal | Observations | Field observations |
| Labridae | Scarus flavipectoralis | Diurnal | Observations | Field observations |
| Labridae | Scarus forsteni | Diurnal | Observations | Field observations |
| Labridae | Scarus frenatus | Diurnal | Observations | Field observations |
| Labridae | Scarus ghobban | Diurnal | Observations | Field observations |
| Labridae | Scarus globiceps | Diurnal | Observations | Field observations |
| Labridae | Scarus niger | Diurnal | Observations | Field observations |
| Labridae | Scarus oviceps | Diurnal | Observations | Field observations |
| Labridae | Scarus psittacus | Diurnal | Observations | Field observations |
| Labridae | Scarus rivulatus | Diurnal | Observations | Field observations |
| Labridae | Scarus rubroviolaceus | Diurnal | Observations | Field observations |
| Labridae | Scarus schlegeli | Diurnal | Observations | Field observations |
| Labridae | Scarus sp | Diurnal | Observations | Field observations |
| Labridae | Scarus spinus | Diurnal | Observations | Field observations |
| Labridae | Stethojulis bandanensis | Diurnal | Observations | Field observations |
| Labridae | Stethojulis strigiventer | Diurnal | Reference | (Nakamura and Tsuchiya 2008) |
| Labridae | Stethojulis trilineata | Diurnal | Observations | Field observations |
| Labridae | Thalassoma amblycephalum | Diurnal | Observations | Field observations |
| Labridae | Thalassoma hardwicke | Diurnal | Observations | Field observations |
| Labridae | Thalassoma lunare | Diurnal | Observations | Field observations |
| Labridae | Thalassoma nigrofasciatum | Diurnal | Observations | Field observations |
| Labridae | Thalassoma quinquevittatum | Diurnal | Observations | Field observations |
| Labridae | Wetmorella albofasciata | Diurnal | Observations | Field observations |
| Lethrinidae | Gymnocranius microdon | Both | Observations | Field observations |
| Lethrinidae | Lethrinus atkinsoni | Both | Observations | Field observations |
| Lethrinidae | Lethrinus harak | Both | Observations | Field observations |
| Lethrinidae | Lethrinus lentjan | Both | Observations | Field observations |
| Lethrinidae | Lethrinus nebulosus | Both | Observations | Field observations |
| Lethrinidae | Lethrinus obsoletus | Both | Observations | Field observations |
| Lethrinidae | Lethrinus olivaceus | Both | Observations | Field observations |
| Lethrinidae | Monotaxis grandoculis | Nocturnal | Reference | (Myers 1999) |
| Lethrinidae | Monotaxis heterodon | Nocturnal | Observations | Field observations |
| Lutjanidae | Aprion virescens | Diurnal | Reference | (Haight et al. 2011) |
| Lutjanidae | Caesio caerulaurea | Diurnal | Observations | Field observations |
| Lutjanidae | Caesio cuning | Diurnal | Observations | Field observations |
| Lutjanidae | Caesio lunaris | Diurnal | Observations | Field observations |
| Lutjanidae | Lutjanus bohar | Both | Observations | field observations |
| Lutjanidae | Lutjanus carponotatus | Both | Observations | field observations |
| Lutjanidae | Lutjanus fulviflamma | Both | Observations | field observations |
| Lutjanidae | Lutjanus fulvus | Both | Observations | field observations |
| Lutjanidae | Lutjanus gibbus | Both | Observations | field observations |
| Lutjanidae | Lutjanus monostigma | Both | Observations | field observations |
| Lutjanidae | Lutjanus quinquelineatus | Both | Observations | field observations |
| Lutjanidae | Lutjanus russellii | Both | Observations | field observations |
| Lutjanidae | Macolor macularis | Both | Observations | field observations |
| Lutjanidae | Macolor niger | Both | Observations | field observations |
| Lutjanidae | Macolor sp | Both | Observations | field observations |
| Lutjanidae | Pterocaesio digramma | Diurnal | Observations | field observations |
| Lutjanidae | Pterocaesio marri | Diurnal | Observations | field observations |
| Lutjanidae | Pterocaesio trilineata | Diurnal | Observations | field observations |
| Monacanthidae | Aluterus scriptus | Diurnal | Observations | field observations |
| Monacanthidae | Paraluteres prionurus | Diurnal | Observations | field observations |
| Mullidae | Mulloidichthys flavolineatus | Nocturnal | Reference | (Holland et al. 1993) |
| Mullidae | Parupeneus barberinus | Both | Observations | field observations |
| Mullidae | Parupeneus ciliatus | Both | Observations | field observations |
| Mullidae | Parupeneus crassilabris | Both | Observations | field observations |
| Mullidae | Parupeneus cyclostomus | Both | Observations | field observations |
| Mullidae | Parupeneus indicus | Both | Observations | field observations |
| Mullidae | Parupeneus multifasciatus | Both | Observations | field observations |
| Muraenidae | Gymnothorax javanicus | Nocturnal | Reference | (Myers 1999; Bshary et al. 2006) |
| Nemipteridae | Pentapodus aureofasciatus | Nocturnal | Observations | Phylogeny & field observations |
| Nemipteridae | Scolopsis bilineata | Both | Reference | (Boaden and Kingsford 2012) |
| Nemipteridae | Scolopsis margaritifera | Nocturnal | Observations | Phylogeny & field observations |
| Nemipteridae | Scolopsis monogramma | Nocturnal | Observations | Relatives & field observations |
| Ostraciidae | Ostracion cubicus | Diurnal | Observations | field observations |
| Ostraciidae | Ostracion meleagris | Diurnal | Observations | field observations |
| Pempheridae | Pempheris oualensis | Nocturnal | Reference | (Fishelson et al. 1971; Myers 1999) |
| Pinguipedidae | Parapercis australis | Diurnal | Reference | (Chase et al. 2018) |
| Pinguipedidae | Parapercis hexophtalma | Diurnal | Observations | field observations |
| Pinguipedidae | Parapercis lineopunctata | Diurnal | Observations | field observations |
| Pinguipedidae | Parapercis queenslandica | Diurnal | Observations | field observations |
| Plesiopidae | Assessor flavissimus | Diurnal | Observations | field observations |
| Plesiopidae | Assessor macneilli | Diurnal | Observations | field observations |
| Pomacanthidae | Centropyge bicolor | Diurnal | Observations | field observations |
| Pomacanthidae | Centropyge bispinosa | Diurnal | Observations | field observations |
| Pomacanthidae | Centropyge vrolikii | Diurnal | Observations | field observations |
| Pomacanthidae | Pomacanthus semicirculatus | Diurnal | Observations | field observations |
| Pomacanthidae | Pomacanthus sexstriatus | Diurnal | Observations | field observations |
| Pomacanthidae | Pomacanthus xanthometopon | Diurnal | Observations | field observations |
| Pomacanthidae | Pygoplites diacanthus | Diurnal | Observations | field observations |
| Pomacentridae | Abudefduf septemfasciatus | Diurnal | Observations | field observations |
| Pomacentridae | Abudefduf sexfasciatus | Diurnal | Observations | field observations |
| Pomacentridae | Abudefduf vaigiensis | Diurnal | Observations | field observations |
| Pomacentridae | Abudefduf whitleyi | Diurnal | Observations | field observations |
| Pomacentridae | Acanthochromis polyacanthus | Diurnal | Observations | field observations |
| Pomacentridae | Amblyglyphidodon curacao | Diurnal | Observations | field observations |
| Pomacentridae | Amblyglyphidodon leucogaster | Diurnal | Observations | field observations |
| Pomacentridae | Amphiprion clarkii | Diurnal | Observations | field observations |
| Pomacentridae | Amphiprion melanopus | Diurnal | Observations | field observations |
| Pomacentridae | Amphiprion percula | Diurnal | Observations | field observations |
| Pomacentridae | Chromis atripectoralis | Diurnal | Observations | field observations |
| Pomacentridae | Chromis atripes | Diurnal | Observations | field observations |
| Pomacentridae | Chromis lepidolepis | Diurnal | Observations | field observations |
| Pomacentridae | Chromis margaritifer | Diurnal | Observations | field observations |
| Pomacentridae | Chromis opercularis | Diurnal | Observations | field observations |
| Pomacentridae | Chromis sp | Diurnal | Observations | field observations |
| Pomacentridae | Chromis ternatensis | Diurnal | Observations | field observations |
| Pomacentridae | Chromis viridis | Diurnal | Observations | field observations |
| Pomacentridae | Chromis weberi | Diurnal | Observations | field observations |
| Pomacentridae | Chrysiptera brownriggii | Diurnal | Observations | field observations |
| Pomacentridae | Chrysiptera caesifrons | Diurnal | Observations | field observations |
| Pomacentridae | Chrysiptera cyanea | Diurnal | Observations | field observations |
| Pomacentridae | Chrysiptera flavipinnis | Diurnal | Observations | field observations |
| Pomacentridae | Chrysiptera rollandi | Diurnal | Observations | field observations |
| Pomacentridae | Chrysiptera talboti | Diurnal | Observations | field observations |
| Pomacentridae | Dascyllus aruanus | Diurnal | Observations | field observations |
| Pomacentridae | Dascyllus reticulatus | Diurnal | Observations | field observations |
| Pomacentridae | Dascyllus trimaculatus | Diurnal | Observations | field observations |
| Pomacentridae | Dischistodus melanotus | Diurnal | Observations | field observations |
| Pomacentridae | Dischistodus perspicillatus | Diurnal | Observations | field observations |
| Pomacentridae | Dischistodus prosopotaenia | Diurnal | Observations | field observations |
| Pomacentridae | Dischistodus pseudochrysopoecilus | Diurnal | Observations | field observations |
| Pomacentridae | Hemiglyphidodon plagiometopon | Diurnal | Observations | field observations |
| Pomacentridae | Neoglyphidodon melas | Diurnal | Observations | field observations |
| Pomacentridae | Neoglyphidodon nigroris | Diurnal | Observations | field observations |
| Pomacentridae | Neopomacentrus azysron | Diurnal | Observations | field observations |
| Pomacentridae | Neopomacentrus cyanomos | Diurnal | Observations | field observations |
| Pomacentridae | Plectroglyphidodon dickii | Diurnal | Observations | field observations |
| Pomacentridae | Plectroglyphidodon johnstonianus | Diurnal | Observations | field observations |
| Pomacentridae | Plectroglyphidodon lacrymatus | Diurnal | Observations | field observations |
| Pomacentridae | Plectroglyphidodon leucozonus | Diurnal | Observations | field observations |
| Pomacentridae | Pomacentrus adelus | Diurnal | Observations | field observations |
| Pomacentridae | Pomacentrus amboinensis | Diurnal | Observations | field observations |
| Pomacentridae | Pomacentrus bankanensis | Diurnal | Observations | field observations |
| Pomacentridae | Pomacentrus brachialis | Diurnal | Observations | field observations |
| Pomacentridae | Pomacentrus chrysurus | Diurnal | Observations | field observations |
| Pomacentridae | Pomacentrus coelestis | Diurnal | Observations | field observations |
| Pomacentridae | Pomacentrus grammorhynchus | Diurnal | Observations | field observations |
| Pomacentridae | Pomacentrus lepidogenys | Diurnal | Observations | field observations |
| Pomacentridae | Pomacentrus magniseptus | Diurnal | Observations | field observations |
| Pomacentridae | Pomacentrus moluccensis | Diurnal | Observations | field observations |
| Pomacentridae | Pomacentrus nagasakiensis | Diurnal | Observations | field observations |
| Pomacentridae | Pomacentrus pavo | Diurnal | Observations | field observations |
| Pomacentridae | Pomacentrus wardi | Diurnal | Observations | field observations |
| Pomacentridae | Premnas biaculeatus | Diurnal | Observations | field observations |
| Pomacentridae | Stegastes apicalis | Diurnal | Observations | field observations |
| Pomacentridae | Stegastes fasciolatus | Diurnal | Observations | field observations |
| Pomacentridae | Stegastes nigricans | Diurnal | Observations | field observations |
| Pomacentridae | Stegastes punctatus | Diurnal | Observations | field observations |
| Priacanthidae | Priacanthus hamrur | Nocturnal | Reference | (Myers 1999) |
| Pseudochromidae | Cypho purpurascens | Diurnal | Observations | field observations |
| Pseudochromidae | Ogilbyina queenslandiae | Diurnal | Observations | field observations |
| Pseudochromidae | Oxycercichthys veliferus | Diurnal | Observations | field observations |
| Pseudochromidae | Pictichromis paccagnellae | Diurnal | Observations | field observations |
| Pseudochromidae | Pseudochromis flammicauda | Diurnal | Observations | field observations |
| Pseudochromidae | Pseudochromis fuscus | Diurnal | Reference | (Bosiger and McCormick 2014) |
| Pseudochromidae | Pseudochromis sp | Diurnal | Observations | field observations |
| Pseudochromidae | Pseudochromis tapeinosoma | Diurnal | Observations | field observations |
| Scombridae | Grammatorcynus bicarinatus | Diurnal | Observations | field observations |
| Scombridae | Scomberomorus commerson | Diurnal | Observations | field observations |
| Scorpaenidae | Parascorpaena mcadamsi | Diurnal | Observations | field observations |
| Scorpaenidae | Scorpaenopsis sp | Diurnal | Observations | field observations |
| Serranidae | Diploprion bifasciatum | Diurnal | Observations | field observations |
| Serranidae | Pseudanthias huchtii | Diurnal | Observations | field observations |
| Serranidae | Pseudanthias squamipinnis | Diurnal | Observations | field observations |
| Siganidae | Siganus argenteus | Diurnal | Reference | (Popper and Gunderman 1975) |
| Siganidae | Siganus corallinus | Diurnal | Observations | field observations |
| Siganidae | Siganus doliatus | Diurnal | Reference | (Brandl and Bellwood 2013) |
| Siganidae | Siganus lineatus | Both | Reference | (Fox and Bellwood 2011) |
| Siganidae | Siganus puellus | Diurnal | Observations | field observations |
| Siganidae | Siganus punctatissimus | Diurnal | Observations | field observations |
| Siganidae | Siganus punctatus | Diurnal | Observations | field observations |
| Siganidae | Siganus sp | Diurnal | Observations | field observations |
| Siganidae | Siganus spinus | Diurnal | Observations | field observations |
| Siganidae | Siganus vulpinus | Diurnal | Observations | field observations |
| Sphyraenidae | Sphyraena flavicauda | Diurnal | Reference | (Sylva 1973) |
| Sphyraenidae | Sphyraena obtusata | Diurnal | Reference | (Sylva 1973) |
| Syngnathidae | Corythoichthys haematopterus | Diurnal | Observations | field observations |
| Syngnathidae | Corythoichthys intestinalis | Diurnal | Observations | field observations |
| Syngnathidae | Corythoichthys sp | Diurnal | Observations | field observations |
| Synodontidae | Saurida gracilis | Diurnal | Observations | field observations |
| Synodontidae | Synodus binotatus | Diurnal | Observations | field observations |
| Synodontidae | Synodus dermatogenys | Diurnal | Observations | field observations |
| Tetraodontidae | Arothron caeruleopunctatus | Diurnal | Observations | field observations |
| Tetraodontidae | Arothron hispidus | Diurnal | Observations | field observations |
| Tetraodontidae | Arothron nigropunctatus | Diurnal | Observations | field observations |
| Tetraodontidae | Arothron stellatus | Diurnal | Observations | field observations |
| Tetraodontidae | Canthigaster papua | Diurnal | Observations | field observations |
| Tetraodontidae | Canthigaster valentini | Diurnal | Observations | field observations |
| Tripterygiidae | Enneapterygius atrogulare | Diurnal | Observations | field observations |
| Tripterygiidae | Enneapterygius similis | Diurnal | Observations | field observations |
| Tripterygiidae | Enneapterygius sp | Diurnal | Observations | field observations |
| Tripterygiidae | Enneapterygius tutuilae | Diurnal | Observations | field observations |
| Tripterygiidae | Ucla xenogrammus | Diurnal | Observations | field observations |
| Zanclidae | Zanclus cornutus | Diurnal | Observations | field observations |

Table S2: Mean estimates of biomass (t ha^-1^) and productivity (g 100m^-2^ day^-1^) with 95% quantile estimates. This table allows for comparisons of the biomass and productivity of the dominant nocturnal reef fish families between sheltered and exposed sites.

|  | Exposed | | Sheltered | |
| --- | --- | --- | --- | --- |
| Family | Biomass  (low 95% - upper 95%) | Productivity  (low 95% - upper 95%) | Biomass  (low 95% - upper 95%) | Productivity  (low 95% - upper 95%) |
| Acanthuridae | 0.166 (0.067-0.251) | 0.196 (0.092-0.296) | 0.137 (0.012-0.251) | 0.132 (0.004 - 0.290) |
| Apogonidae | 0.006 (0.001-0.015) | 0.069 (0.008-0.266) | 0.201 (0.000-0.831) | 1.147 (0.008 - 2.826) |
| Haemulidae | 0.105 (0.026-0.204) | 0.035 (0.006-0.081) | 0.452 (0.000-2.385) | 0.168 (0.000 - 0.896) |
| Lethrinidae | 0.030 (0.010-0.056) | 0.019 (0.011-0.033) | 0.095 (0.020-0.409) | 0.069 (0.009 - 0.268) |
| Lutjanidae | 0.088 (0.012-0.168) | 0.063 (0.014-0.135) | 0.398 (0.070-1.503) | 0.180 (0.062 - 0.344) |
